# Isatin-1,8-Naphthalimide Hydrazones: A Study of Their Sensor and ON/OFF Functionality

**DOI:** 10.3390/molecules24030397

**Published:** 2019-01-22

**Authors:** Pavol Tisovský, Miroslav Horváth, Klaudia Csicsai, Jana Donovalová, Juraj Filo, Marek Cigáň, Róbert Sokolík, Gabriela Addová, Anton Gáplovský

**Affiliations:** Faculty of Natural Sciences, Institute of Chemistry, Comenius University, Ilkovičova 6, Mlynská dolina CH-2, SK-842 15 Bratislava, Slovakia; mirek.horvath@gmail.com (M.H.); klaudia.jakusova@uniba.sk (K.C.); jana.donovalova@uniba.sk (J.D.); juraj.filo@uniba.sk (J.F.); marek.cigan@uniba.sk (M.C.); robert.sokolik@uniba.sk (R.S.); gabriela.addova@uniba.sk (G.A.) anton.gaplovsky@uniba.sk (A.G.)

**Keywords:** on/off switches, anion sensors, intramolecular interaction, UV-Vis spectroscopy

## Abstract

Five novel hydrazones derived from substituted isatins were synthesized as potential anion sensors. Using UV-VIS, FTIR, NMR and fluorescence spectroscopy, these compounds’ tautomeric equilibrium and *Z*-*E* photoisomerization were studied in DMF and CHCl_3_, depending on the hydrazone concentrations, the presence of basic anions and light stimulation. Anion recognition aspects (PF_6_^−^, HSO_4_^−^, Br^−^, Cl^−^, NO_3_^−^, F^−^ and CH_3_COO^−^) and these receptors’ detection limits were also studied. We also tested the light-stimulated ON-OFF functionality of these compounds in the presence or absence of these anions.

## 1. Introduction

Hydrazones belong to a group of chemical compounds whose photochromic properties are a prerequisite for their practical application as binary switches. From a practical point of view, they are very attractive molecules, with the switch function, where the light is used on its input and output. This type of molecules represents the simplest switch that can distinguish between two states: ON-OFF. To perform more complicated logic operations, it is necessary to provide a complex system. This system consisting of two or more suitably interconnected simple molecular switches either a system, which consists of one switch with multiple inputs and outputs. The photochromic molecules ability “to function” as molecular switches and to communicate with other chromophores create conditions for on Boolean’s logic working photonic gates formation. In our previous works the isatin *N^2^*-diarylhydrazone [1] or arylhydrazones [2,3] and their deprotonated forms were examined with the aim to use them as organic material for electronics or anion sensors. The UV-Vis spectra of these compounds are similar. Depending on the structure they have the absorption maximum in the wavelength range from 390 nm to 430 nm. For all previously studied hydrazones, we have used their colorimetric properties or changes in their colorimetric parameters. Due to very small fluorescence quantum yield of these compounds the fluorescence was not used. Therefore, our goal was to prepare isatin hydrazones with sufficiently high fluorescence intensity and at the same time sufficiently large the fluorescence intensity change, as response to the stimulus. Naphthalimide is an easily modifiable structural fragment that is the part of many, more complex organic compounds and organic functional materials. For example, naphthalimide derivatives have been used as supramolecular units to study photo-induced electron transfer (PET) [4,5] as fluorescence bioprobes [6,7], laser dyes [8], fluorescence brighteners [9] and sensors [10,11,12,13]. Due to their high application potential, their photochemical and thermal stability was studied in detail [14,15,16]. 1,8-naphthalimide hydrazones are conjugated organic compounds with planar structure [17]. The 1,8-naphthalimide system is characterized by a strong positive quadrupole moment, that gives these molecules the opportunity to interact with anions through non-covalent anion-π interaction [18]. Cheshmedzhieva et al. have found that 1,8-naphthalimide arylhydrazones with electron-donor groups increase fluorescence quantum yields [19]. Through these groups, it is possible to set up emission wavelengths up to the red spectrum range. The isatin hydrazone lowest excited state is n-π*-electron state which—after its deactivation and photoisomerization—effectively competes with fluorescence [20]. Therefore, isatin hydrazones have very low fluorescence quantum yield. We tried to increase their excited state deactivation probability by fluorescence by introducing the 1,8-naphthalimide fragment into the isatin hydrazones structure. We prepared five new isatin 1,8-naphthalimide hydrazones.

## 2. Results and Discussion

### 2.1. Synthesis

**Scheme 1:**
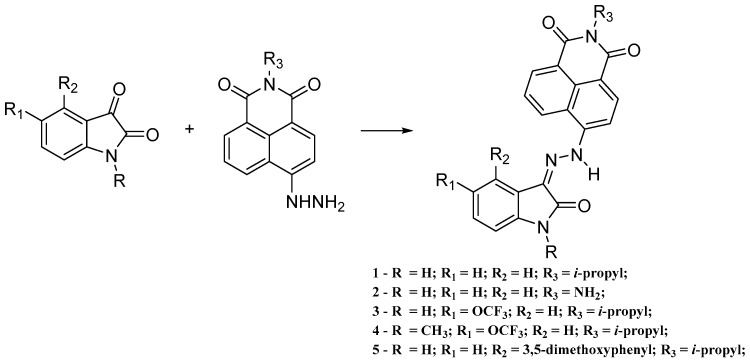
Synthesis of compounds **1** to **5**.

Compounds **1**–**5** were prepared by condensation reaction of the corresponding isatin with hydrazine (Scheme 1).

The corresponding products were isolated in high yields (71–99%). From the reaction mixture we isolated desired products with the *Z*-isomer configuration. Intramolecular hydrogen bond contributes to the *Z*-isomer stabilization. Compound **5** was designed so that the *E*-isomer was stabilized by the donor-acceptor interaction. Based on measurements, we found that by the synthesis the *E*-isomer was also formed. This isomer presence in the reaction mixture depends on the reaction conditions. In chloroform, the *E:Z* ratio is even 1.5:1, what demonstrates, that CT (charge transfer) interaction increases the *E*-isomer stability. 

### 2.2. UV-VIS Spectra

**Figure 1:**
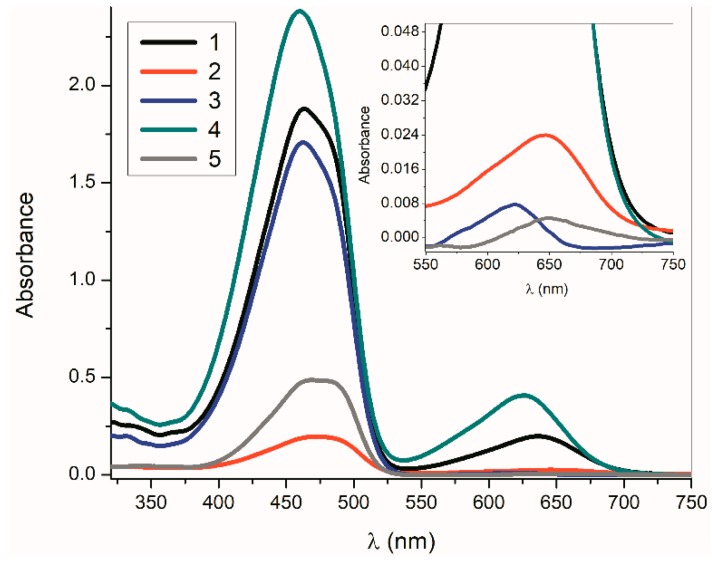
The studied isatin hydrazones **1**, **3**–**5** (5 × 10^−5^ mol.dm^−3^) and **2** (saturated solution) UV-Vis spectra in DMF.

In the studied hydrazone molecules the 1,8-naphthalimide moiety electron deficient character is significantly affecting the charge distribution. It also appeared in the ultraviolet-visible (UV-Vis) spectra (Table 1). Compared to the isatin *N^2^*-diaryl hydrazones or arylhydrazones these compounds have a long absorbent band, which is batochromically shifted at least about 30 nm [1,2]. The maximum band position is at 460 nm and depends a little on solvent polarity. These compounds also absorb in high dielectric constant solvents (dimethylformamide (DMF), dimethyl sulfoxide (DMSO)) in the range from 550 nm to 700 nm (Figure 1). In this spectrum region absorption bands are weak. Their intensity depends on their structure and hydrazone concentration.

**Figure 2:**
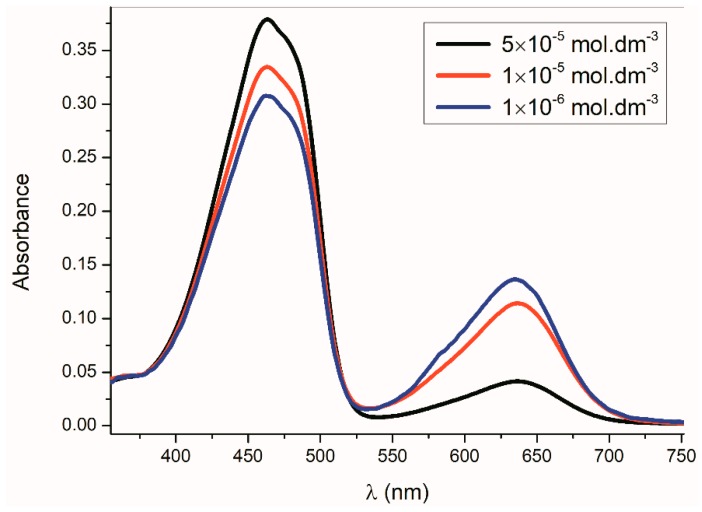
Hydrazone **1** UV-Vis spectra concentration dependence in DMF (recalculated).

With an increasing hydrazone concentration, the band intensity decreases at 630 nm and at the same time the band intensity increases at 460 nm (Figure 2). Concentration dependence confirms the donor-acceptor intermolecular interactions existence that contribute to the hydrazone structure stabilization [21].

### 2.3. Tautomeric Equilibrium Z-E and E-Z Isomerization

**Figure 3:**
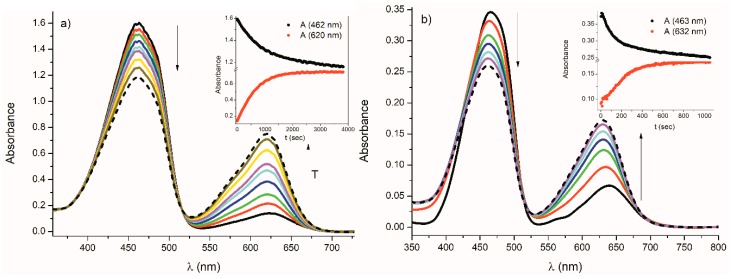
UV-Vis spectra change: (**a**) hydrazone **3** (5 × 10^−5^ mol.dm^−3^) at temperature 50 °C and (**b**) hydrazone **1** (10^−5^ mol.dm^−3^) at temperature 80 °C as the time function in DMF (in set: kinetic course of reaction isothermal change).

The absorption maxima ratio reversible temperature dependency at 460 nm and 630 nm (Figure 3) confirms the chemical equilibrium existence between the hydrazone and the substance, that absorbs at 630 nm. The UV-Vis spectra concentration dependence was observed, e.g., at 3-(2-phenylhydrazono)indolin-2-one, too [2].

**Figure 4:**
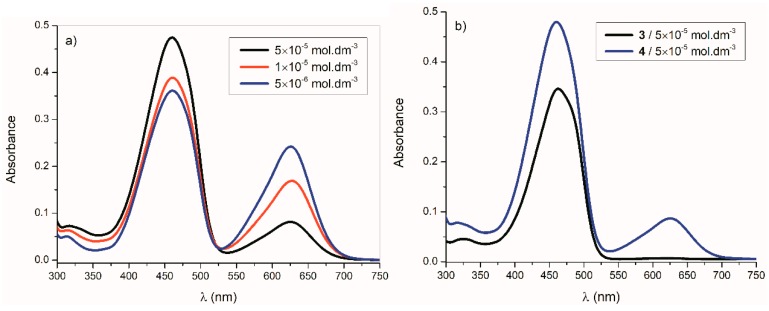
Hydrazone **3** and **4** UV-Vis spectra in DMF, (recalculated). (**a**) hydrazone **4** the concentration effect; (**b**) the isatin methylation effect on the UV-Vis spectrum.

**Scheme 2:**
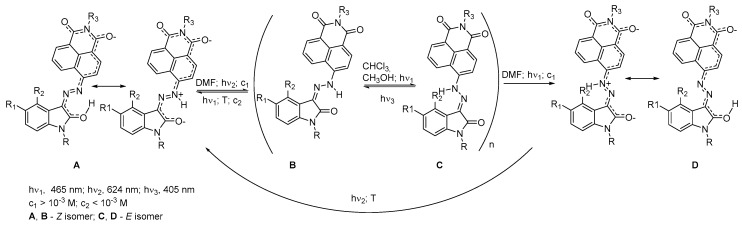
The hydrazone concentration, light, temperature and solvent polarity effect on the studied compounds equilibrium reactions.

In these works [2,3], we have shown that the lowest energy band resulting from the hydrazone concentration decreasing, arises because of the cyclic hydrogen bond disappearance between two isatin hydrazone moieties. In the case of isatin 1,8-naphthylimide hydrazones this was not observed. The isatin NH hydrogen substitution for the methyl group, does not prevent of the band formation at 630 nm. Methylation leads to increase of the band intensity at 630 nm as compared to an analogous non-methylated compound (Figure 4a,b) 160 nm difference in the hydrazones between **B** maximum positions (about 460 nm) and the form that absorbs at 630 nm indicates the large charge transfer in the molecule (Scheme 2).

**Figure 5:**
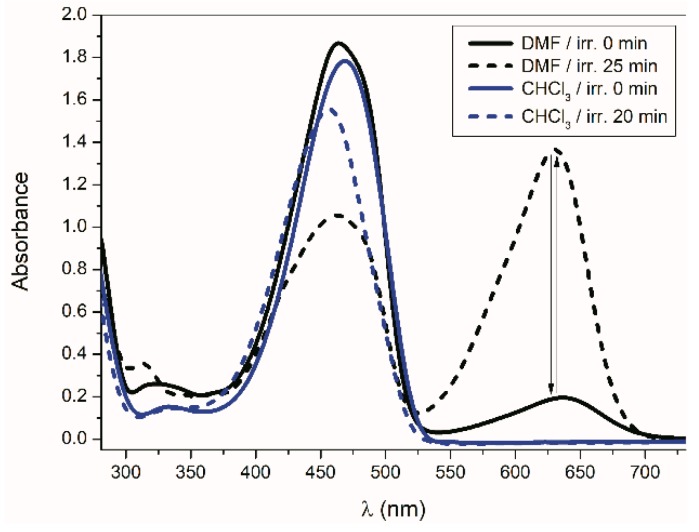
Compound **1** UV-Vis spectra change in DMF and CHCl_3_ (5 × 10^−5^ mol.dm^−3^) during irradiation with wavelength 465 nm.

**Figure 6:**
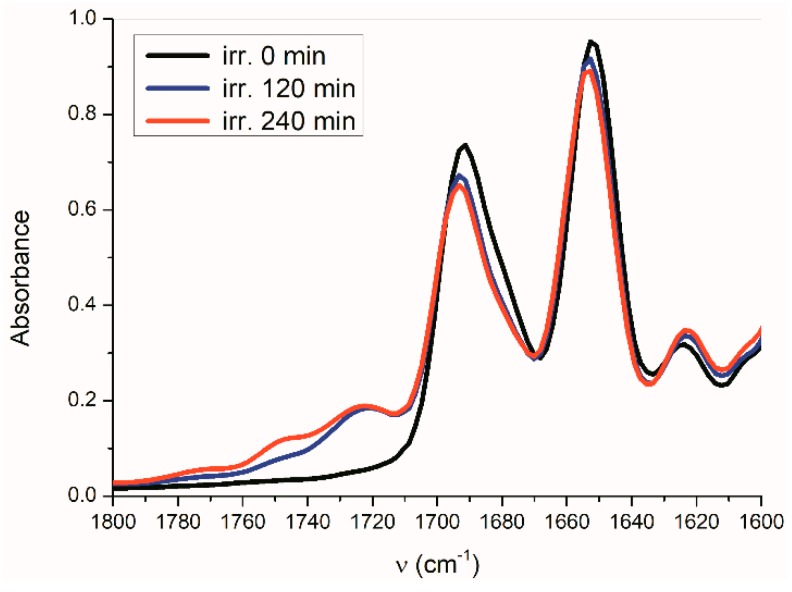
Compound **4** (1 × 10**^−^**^3^ mol.dm^−3^) FTIR spectra during irradiation (λ = 525 nm) in CHCl_3_.

We assigned structures **A** and **D** to the compound absorbing in UV-Vis spectra at 630 nm. These structures are stabilized by aprotic solvents with the sufficiently high dielectric constant (e.g., DMSO, DMF, acetonitrile). This conclusion is consistent with spectral measurements as well as with the theoretical calculations results [22]. The structures **A** and **D** C=N charge density is lower than the **B** and **C**. Intramolecular hydrogen bond also disappeared in the structure **D**. Extinction or weakening of an intramolecular molecular hydrogen bond, the stability of the molecule **D** decreases that—after irradiation with the light 465 nm or with the temperature—it changes to structure **A**. This was proved by ^1^H-NMR and High-performance liquid chromatography (HPLC). In nonpolar (CHCl_3_) and polar protic solvents (CH_3_OH), structures **A** and **D** do not form, respectively, if they form, their concentration is below the used spectral methods detection limit. After studied hydrazones irradiation in these solvents, the hydrazones typical photochemical reaction i.e., the reversible geometric isomerisation around the C=N bond occurs (Figure 5; supplementary Figures S1 and S2). The *E* and *Z* studied compound isomers UV-Vis spectra show a slight difference. From Figure 6, is evident that during the *Z*⇄*E* isomerisation, the new bands at the higher frequencies corresponding to the *E*-isomer are formed in spectrum of compound **4** in the carbonyl region in the Fourier transform infrared spectra (FTIR). This is mainly caused by the intramolecular hydrogen bond disappearance in the molecule and the *E* isomer planarity decreases.

In Table 2. are shown the *Z* and *E* isomer relative abundance in the equilibrium state after *Z*-isomer (5 × 10^−5^ mol·dm^−3^) irradiation with 465 nm in CHCl_3_ and DMF (determined by HPLC). 

**Figure 7:**
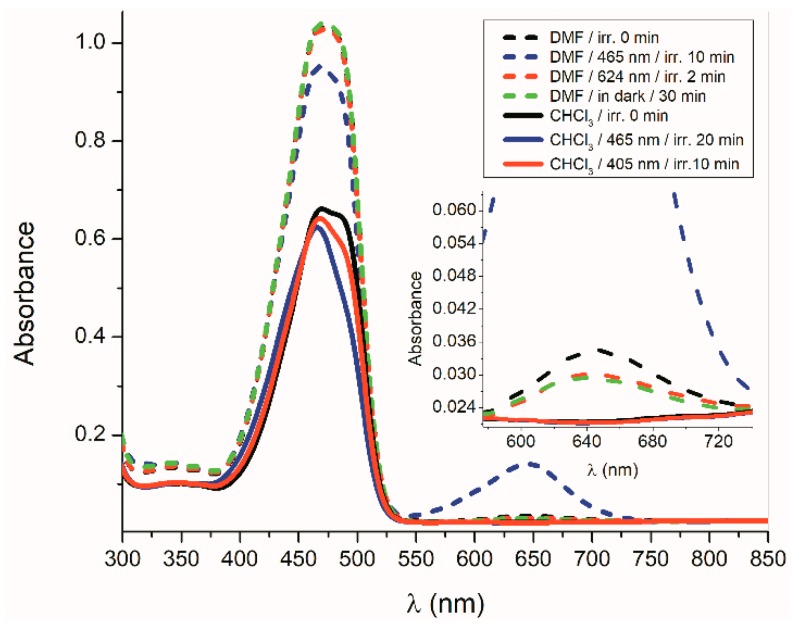
Compound **5** (1 × 10^−4^ mol·dm^−3^) UV-Vis spectra change during photochemical isomerisation in DMF and CHCl_3_.

At all the studied compounds in CHCl_3_ the *E*-isomer abundance in the photostationary state depends on their coefficients ε_λirr_ differences corresponding to the *E* and *Z* isomers. From the data in Table 2 for the *Z*-isomers irradiation is evident that even in DMF, *E*-isomers are formed, however, the *E*-isomer ratio is low compared to that in CHCl_3_. This is due to either thermal and photochemical stability or **A** and **D** reactivity (Scheme 2). For this reason, the *E*-isomer abundance is low, in the studied hydrazones synthesis in aprotic solvents with a high dielectric constant. This was confirmed in the compound **5** synthesis in DMF where the *E*-isomer abundance was negligible but in CHCl_3_ it was up to ~60%. Different photoreactivity in DMF and CHCl_3_ can be seen very well in Figure 7. In hydrazones UV-VIS spectra in polar aprotic solvents (DMF, DMSO) during irradiation (465 nm), was observed the band formation at 630 nm (Figure 5 and Figure 7; supplementary Figure S3).

**Figure 8:**
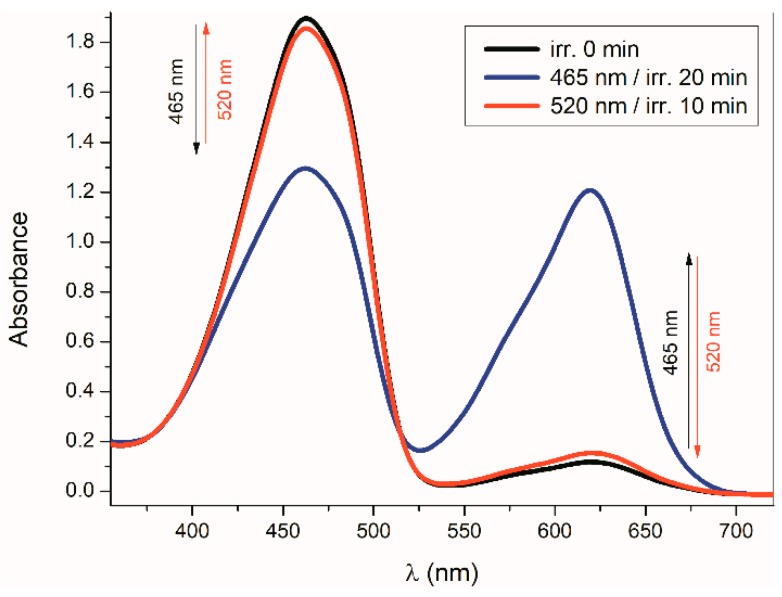
Compound **3** (1 × 10^−4^ mol·dm^−3^) UV-Vis spectra change during irradiation, alternating by turns with different wavelength lights (465 nm a 624 nm) in DMF.

This band is the same as it was observed at the described concentration dependence. The change in UV-Vis spectra is reversible. Using light with λ > 520 nm band intensity at 630 nm decreases with the simultaneous band intensity increase at 460 nm (Figure 8; supplementary Figure S3).

**Figure 9:**
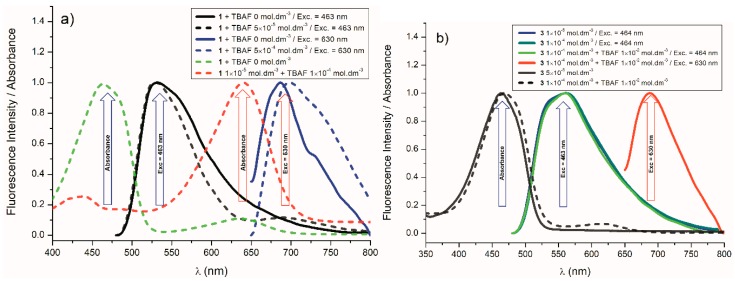
Normalized fluorescence and absorption spectra (**a**) hydrazone **1** (DMF) (**b**) hydrazone **3** (CHCl_3_) in the presence and absence of F^−^ ions.

**Figure 10:**
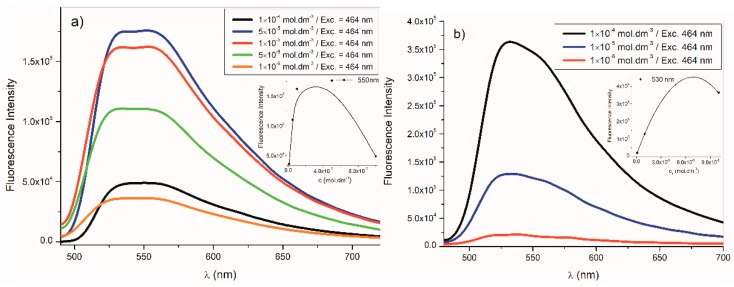
Concentration fluorescence quenching of hydrazone **1**, (**a**) in CHCl_3_, (**b**) in DMF after excitation λ_exc._ = 464 nm.

Compared to other isatin arylhydrazones the 1,8-naphthylimide fragment presence in the studied hydrazones increased their fluorescence quantum yields. For compound **1** the highest fluorescence quantum yield in DMF and methanol was observed (Table 3). The tautomeric equilibrium existence was also confirmed by fluorescence measurements (Figure 9). We also observed two fluorescence bands dependent on the excitation wavelength (460 nm and 630 nm). One at λ_exc._ = 460 nm with the maximum fluorescence around 550 nm (hydrazone form **B** or **C**) and the other at λ_exc._ = 630 nm with the maximum around 690 nm (tautomeric form **A** and **D**) (Scheme 2). The both maxima positions relatively slightly depend on the hydrazones structure and on the solvent polarity (Table 1). Both fluorescent bands are the corresponding absorbent band mirror image (Figure 9). The fluorescence intensity at 550 nm in CHCl_3_ and DMF is concentration dependent (Figure 10).

In DMF is the concentration quenching less effective. In both solvents this concentration quenching is probably caused by intermolecular Coulombic interactions that stabilize the hydrazone form **B** or **C** (Scheme 2). The concentration changes influences fluorescence spectra at λ_exc._ = 465 nm and UV-Vis spectra in the same way.

**Figure 11:**
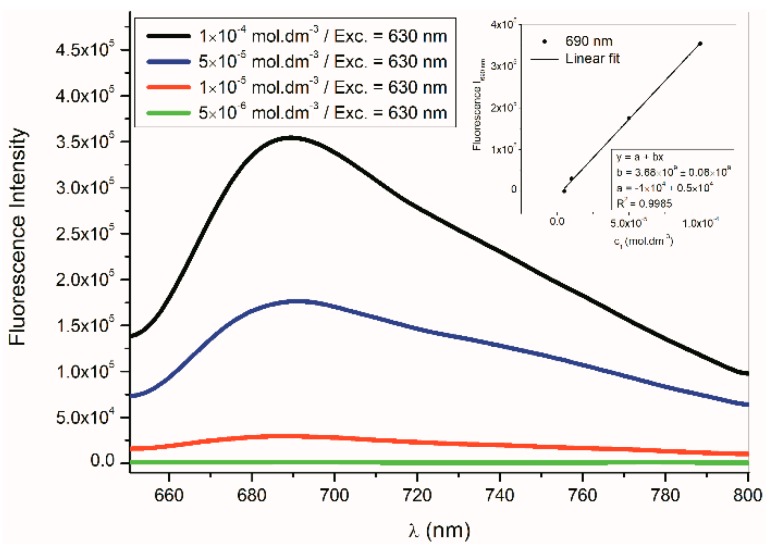
Compound **1** fluorescence intensity change with the concentration change at excitation 630 nm in DMF.

**Figure 12:**
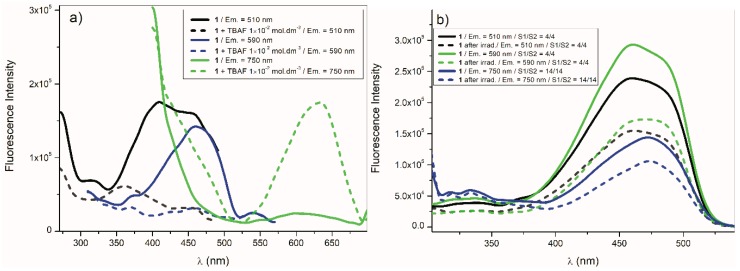
Excitation spectra non-irradiated and irradiated (λ_exc._ = 465 nm) compound **1** in (**a**) DMF and (**b**) CHCl_3_ (λ_em._ = 510 nm, 590 nm and 750 nm).

Fluorescence at 690 nm (tautomeric form **D** or **A**) varies linearly and is not influenced by concentration quenching (Figure 11). Tautomeric equilibrium changes and the F^−^ ions effect on it can also be monitored by excitation spectra (Figure 12). 

In the spectral region from 350 to 525 nm using the excitation spectra, rather than the UV-Vis spectra in DMF it is possible to distinguish the individual compounds involved in the equilibrium state. This change is likely corresponding to the geometric changes in the hydrazone molecule. We obtained three fluorescence lifetimes by mathematical fitting of the fluorescence lifetime curve (by three-exponential function). From the data in Table 3 and Figure 1 is evident the correlation between the electronic states abundance with lifetime τ_2_ percentage the studied hydrazone tautomeric forms **A** or **D** abundance in DMF (Scheme 2). 

After excitation at 630 nm we did not observe this tautomeric form fluorescence for compound **5**. In CHCl_3_ where the tautomeric equilibrium is shifted to hydrazoforms we have not observed radiative excited states with the lifetime τ_2_. Based on these findings we assigned the lifetime τ_2_ to the tautomeric forms **A** or **D**. The main radiative deactivation pathways of compounds **3** to **5** (Table 3) is the process with the longest lifetime τ_3_. These are compounds which are substituted on the isatin moiety. This substitution leads to more consistent isatin nitrogen free electron pair involvement into the conjugation with the isatin π-electron system [23]. The isatin aromatic system charge density increasing decreases the tautomeric process probability. It supports hydrazone form these compounds existence. Based on the solvent and the concentration effect on tautomeric equilibrium the lifetime τ_3_ was assigned to the hydrazone aggregated forms and τ_1_ monomeric form [24]. Similar slightly lower lifetime values for other 1,8-naphthalimide hydrazones were measured in works [22,25].

### 2.4. The Ions Effect on UV-Vis and Fluorescence Spectra of Isatin 1,8-Naphthalimide Hydrazones—Anion Sensors

**Figure 13:**
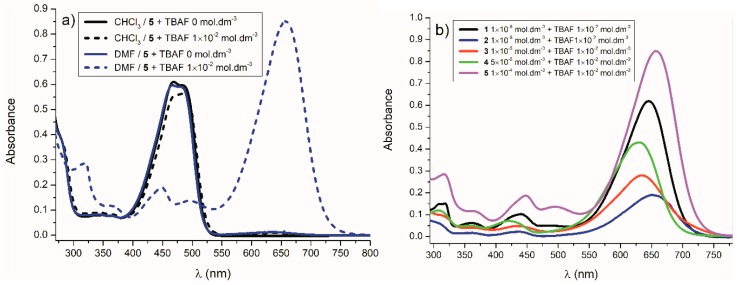
Hydrazone UV-Vis spectrum: (**a**) the solvent effect (DMF, CHCl_3_) and fluoride ions (tetrabutylammonium fluoride (TBAF)) effect on the UV-Vis spectrum of **5** (1 × 10^−4^ mol·dm^−3^); (**b**) the hydrazone structure and TBAF effect on the UV-Vis spectrum of compounds **1**–**5** in DMF.

**Scheme 3:**
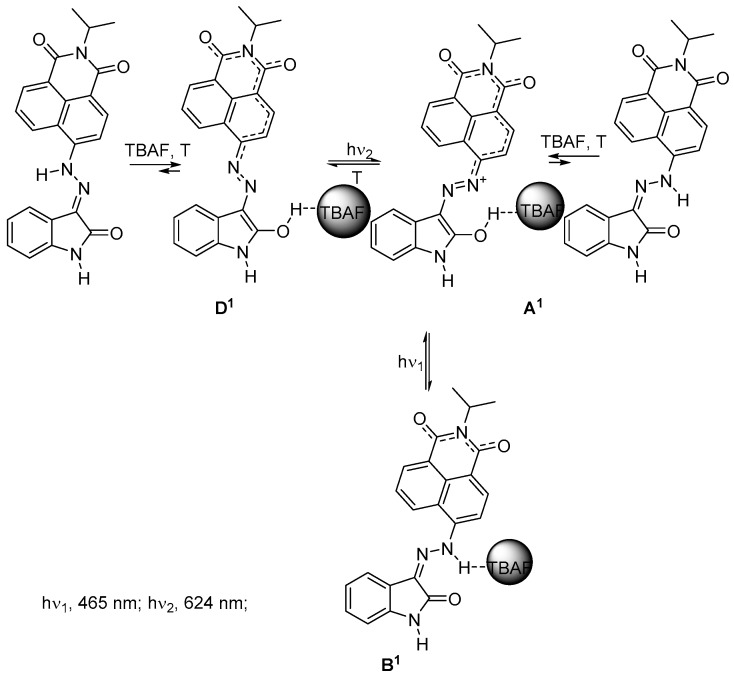
TBAF effect on equilibrium and photochemical reactivity of studied compounds.

**Figure 14:**
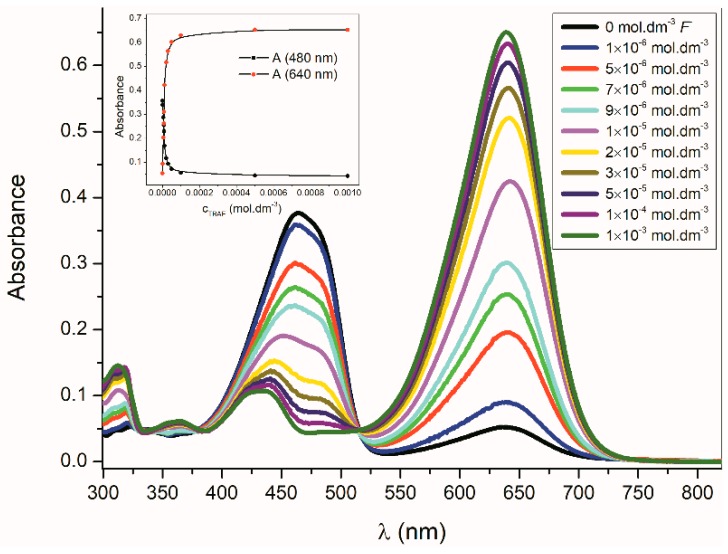
TBAF effect on UV-Vis spectra of compound **1** (1 × 10^−5^ mol·dm^−3^) in DMF.

Studied hydrazones in the presence of fluoride, chloride and acetate ions in DMF provide coloured solutions similar to those in works [2,3]. These ions presence in the solution increases the dielectric medium properties and at the same time these ions interact with NH hydrazone hydrogen. Due to these processes the charge transfer occurs in the hydrazones that appears in new absorption band formation with the maximum around 630 nm in UV-Vis spectra (Scheme 3) which is practically identical with the tautomeric forms **A** and **D** bands (Scheme 2 and Figure 13). By the given hydrazone concentration the band intensity at 630 nm depends on the ions concentration in solution (Figure 14, Figure S4). 

After ions addition to the hydrazone *Z*-isomer solution an intramolecular hydrogen bond is weakened by an anionic interaction and the tautomeric form **A^1^** is formed.

**Figure 15:**
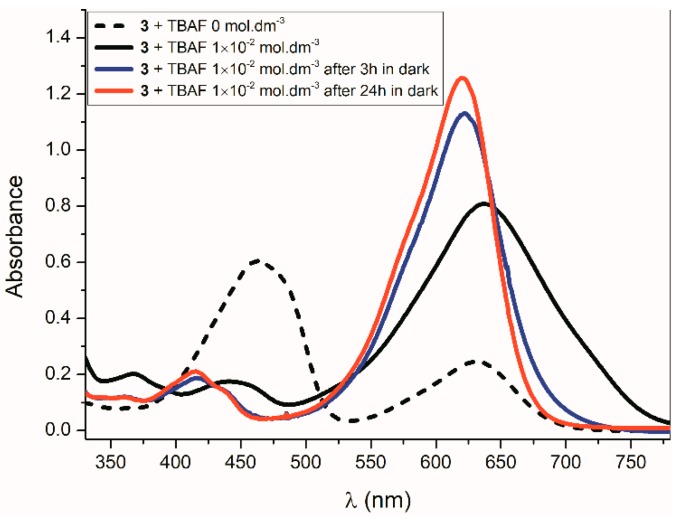
Compound **3** (1 × 10^−5^ mol·dm^−3^) UV-Vis spectrum thermal change in the presence of TBAF (1 × 10**^−^**^2^ mol·dm^−3^) in DMF.

Within a few minutes after the ion addition to the solution on UV-Vis spectra the hypsochromic shift (approximately 15 nm) of the originally formed band at 630 nm is observed (Figure 15). 

During NMR experiment in CDCl_3_ and DMSO (supplementary Figures S5 and S6), after TBAF addition tautomeric change from **A^1^** to **D^1^** was observed according to proposed mechanism in Scheme 3. This change is observed as a time dependent signal intensity change and is illustrated in Figures S5 and S6 as a black arrow for representing signals. Signal intensity decrease belongs to **A**^1^ and signal intensity increase to **D^1^** form respectively. These results are in good agreement with those ones observed by UV-Vis spectroscopy (Figure 15). Cl^−^ ions (tetrabutylammonium chloride, TBACl) have significantly smaller effect on the studied hydrazones UV-Vis spectrum than F^−^ or CH_3_COO^−^ ions.

**Figure 16:**
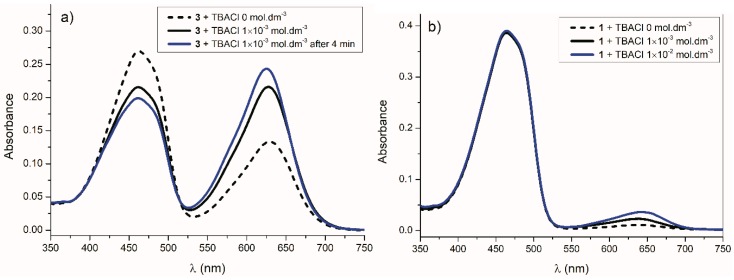
Effect of Cl^−^ ions (TBACl) on UV-VIS spectra (**a**) compound **3** (1 × 10^−5^ mol·dm^−3^); (**b**) compound **1** (1 × 10^−5^ mol·dm^−3^) in DMF.

Depending on the hydrazone structure this effect is lesser than 200–1000 times (Figure 16). This is due to the lower Cl^−^ ions basicity [26]. The size of Cl^−^ ions impact on the UV-Vis spectrum (band intensity at 630 nm) depends on the hydrazone structure or the hydrazone NH hydrogen acidity, therefore it maintains the same order as we observed for F^−^ ions. Cl^−^ ions have the largest effect on compound **3** (Figure 16). Similar to F^−^ ions the thermal reaction between structures **A^1^** and **D^1^** (Scheme 3) was observed in the presence of Cl^−^ ions too, which causes the band hypsochromic shift at 630 nm around approximately 5 nm. 

**Figure 17:**
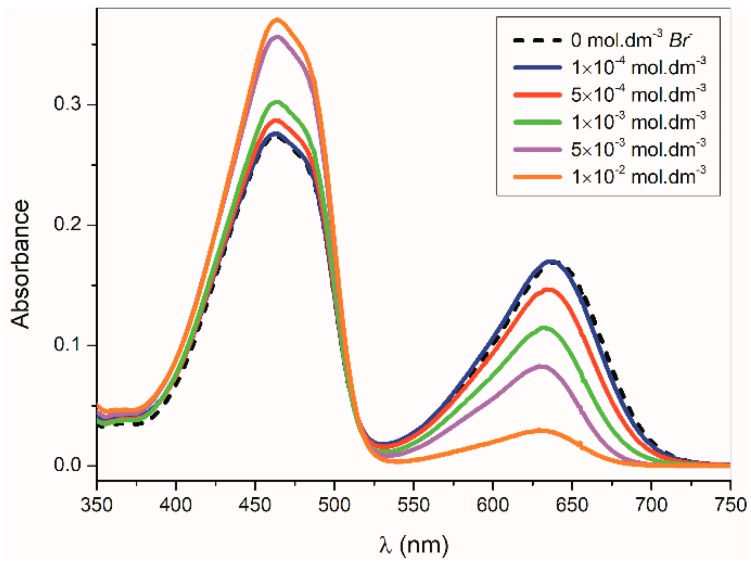
Br^−^ ions (TBABr) effect on UV-VIS spectrum of compound **1** (1 × 10^−5^ mol·dm^−3^) in DMF.

**Figure 18:**
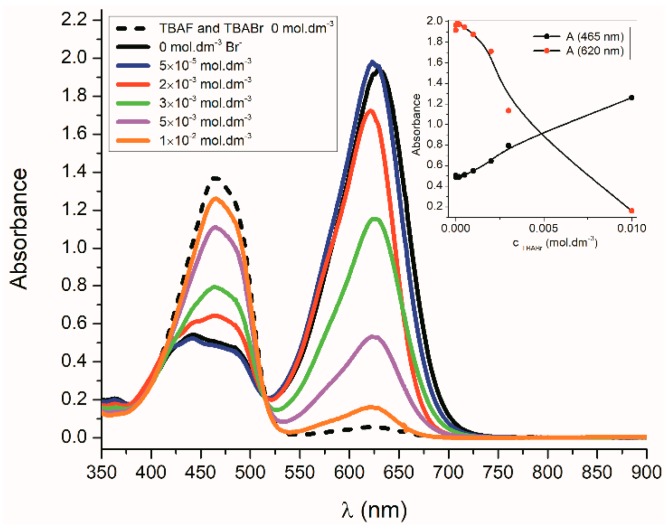
TBABr concentration effect on compound **3** UV-Vis spectra (5 × 10^−5^ mol·dm^−3^) in the presence of TBAF (5 × 10^−5^ mol·dm^−3^) in DMSO.

The influence of tetrabutylammonium bromide (TBABr) on the tautomeric equilibrium can be explained by different relative anion basicity (Figure 17). TBABr shifts the tautomeric equilibrium towards hydrazone form **B** (scheme 2). TBABr at the hydrazone concentrations at which the band can be observed at 630 nm in the UV-Vis spectra decrease this band intensity. A similar effect has TBABr also on the UV-Vis spectrum of compound **3** solution (1 × 10^−5^ mol·dm^−3^) with TBAF (1 × 10^−5^ mol·dm^−3^) (Figure 18).

The band intensity decreases about 72% at 630 nm (tautomer **A^1^**) at the TBABr concentration (5 × 10^−3^ mol·dm^−3^) equal to 100 times the TBAF concentration. Further decrease in absorbance is achieved (about 20% at 630 nm) by increasing the TBABr concentration to 1 × 10^−2^ mol·dm^−3^ (200-fold excess). TBABr decreases the F^−^ ability ions to interact with hydrazone NH hydrogen. We assume that TBABr electrostatically decreases the F^−^ ions basicity in the way that these ions are no longer able to compete with intramolecular hydrogen bond in the studied *Z*-isomers. We estimated the studied hydrazones pKa value based on experiments with ions PF_6_^−^, HSO_4_^−^, Br^−^, Cl^−^, NO_3_^−^, F^−^ and CH_3_COO^−^ and H-Bond Acceptor Parameters for Anions (β) [26]. The pKa value is within the range: 0.9 < pKa < 1.8 i.e., between values of pKa Br^−^ and Cl^−^ ions in DMSO. All studied hydrazones are selective sensors for fluoride and acetate anions.

From the data in Table 4 (detection and quantification limits) is evident that compounds **3** and **4** are—compared to the published data [27]—so far very good F^−^ ion sensors. Relatively high sensitivity of studied hydrazones also to the acetate ions presence (Table 4) decreases their selectivity or versatility as sensors to F^−^ ions. Detection limit values (Table 4) suggest that the apparent association constants K_ass_ studied 1,8-naphthalimide derivatives will acquire high values in the presence of strongly basic F^−^ and CH_3_COO^−^ anions in DMF. This was confirmed by the titration data non-linear fitting (absorption dependence on F^−^ and CH_3_COO^−^ ions concentration at 630 nm, 1: 1 complex sensor anion). Thus, the obtained K_ass_ constants acquire very high, unrealistic values (for example supplementary Figure S7). Binding constants K_ass_ accurate determination was prevented by the titration plot steep curvature. The unrealistic K_ass_ constants might be caused by the titration process complexity. The titration process consists of several processes whose application degree depends on the ion concentration for the titration used. The ions presence also reflects in the studied compound fluorescence spectra. Tautomeric equilibrium effects the fluorescence (Scheme 3).

**Figure 19:**
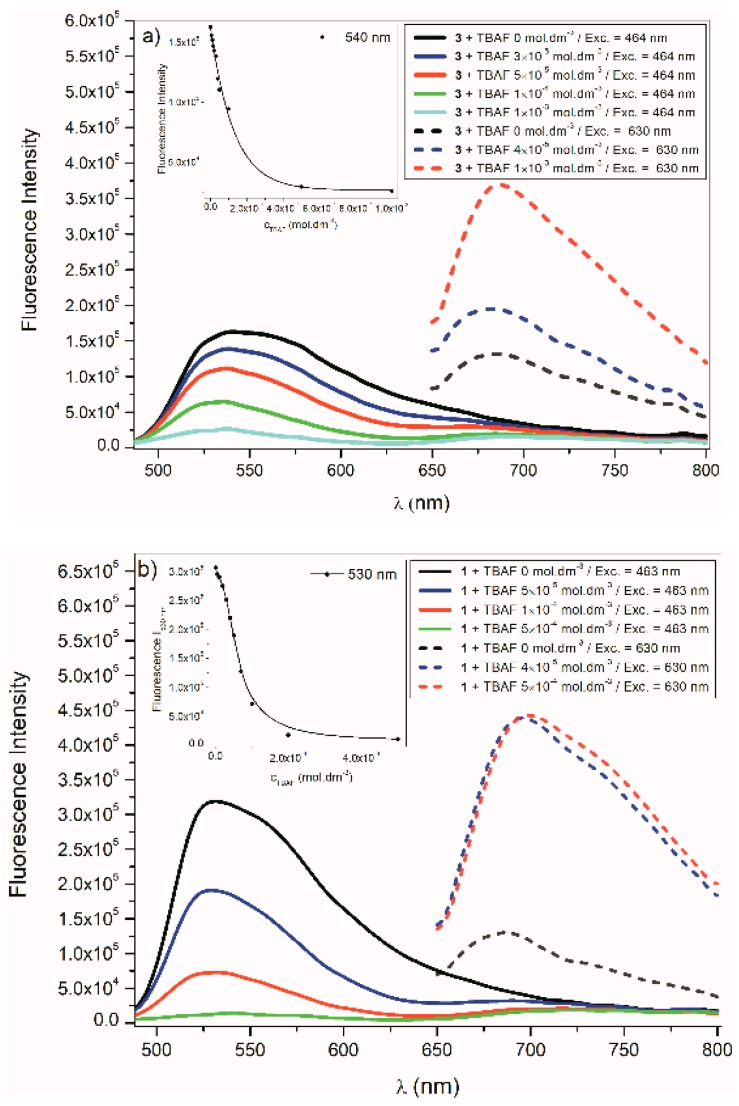
(**a**) compound **3** (5 × 10^−5^ mol·dm^−3^) and (**b**) compound **1** (5 × 10^−5^ mol·dm^−3^) fluorescence spectra change in the presence of F^−^ ions in DMF (λ_exc_ = 465 nm and 630 nm).

After excitation at 465 nm, the hydrazone form **B** or **C** fluorescence intensity decreases (Scheme 2) with maximum at 550 nm, in the F^−^ ions presence which shift the tautomeric equilibrium towards forms **A^1^** or **D^1^** (Figure 19). In the F^−^ ions presence equilibrium shift towards the tautomeric form **A^1^** or **D^1^** after excitation (630 nm) is manifested by increase fluorescence intensity with maximum at 690 nm (Figure 19).

### 2.5. Light-Stimulated Isatin 1,8-Naphthalimide Hydrazones ON/OFF Properties or the System Isatin 1,8-Naphthalimide Hydrazone + Anions

**Figure 20:**
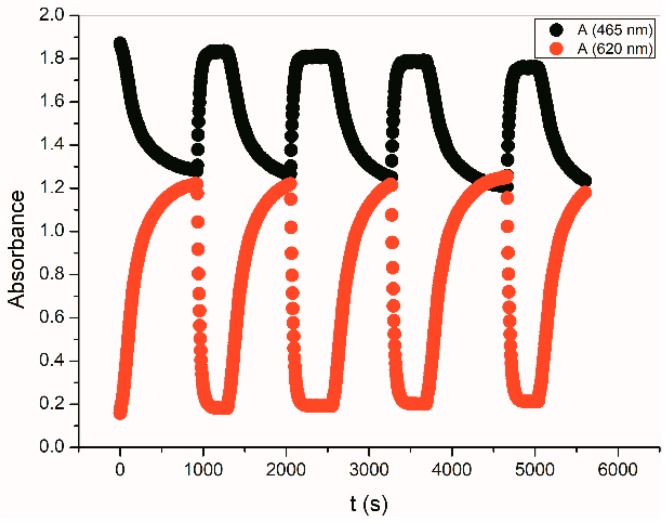
Absorption changes kinetic recording of absorption maxima during compound **3** irradiation alternately with light of different wavelength (465 nm and 630 nm) in DMF.

In the introduction to this discussion we have mentioned that the tautomeric change occurring between forms **A** and **D** (Scheme 2) in solvents such as DMF or DMSO is reversible. The reaction direction is controlled by light with different wavelength (Figure 8). This tautomeric equilibrium extent depends on the hydrazone structure [2,3], the environment dielectric properties and the hydrazine concentration or intermolecular interactions (association, etc.) that stabilize some of tautomeric forms. If light as the switching stimulus and as the response colorimetric or fluorescence change was used by the structure modification we can relatively easily adjust the switching spectral region, e.g., UV-Vis, Vis-Vis, and so on. For the given structure it is almost always necessary to “adjust” the conditions, these molecule surroundings to reach the ON/OFF switching reliable in sufficient range and fast enough. In this work the hydrazones structure has been designed so that the stimulus and colorimetric respond were in visible spectrum area i.e., Vis-Vis switching. Tautomeric equilibrium can be controlled by different energy means (465 nm and 624 nm) (Figure 20). The reversibility, the stability of this process, the large differences in absorption maxima in the Vis spectra and the absorbance values give this hydrazone the potential for practical use as the signal switch. 

**Figure 21:**
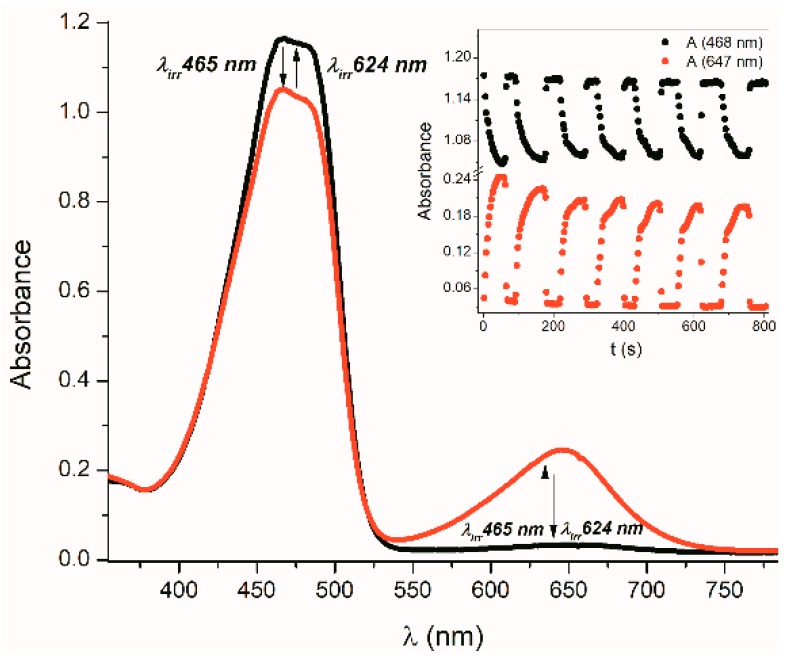
Compound **5** (5 × 10^−5^ mol.dm^−3^) UV-Vis spectra change in the presence of TBAF (2 × 10^−5^ mol·dm^−3^) during alternating irradiation with the wavelength light at 465 nm or 624 nm in DMF.

**Scheme 4:**
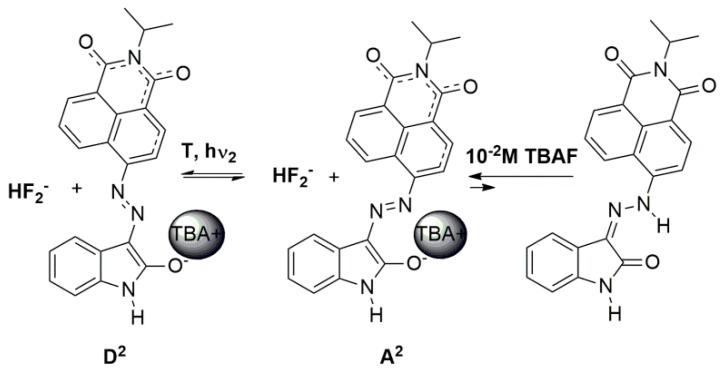
NH hydrazone hydrogen cleavage at high TBAF excess and the formed product reactivity.

The compound **5** tautomeric equilibrium (Scheme 2) is almost completely shifted to the hydrazone side (Figure 1). Based on experimental measurements the hydrazones behaviour in the ions presence is described in Scheme 3 and Scheme 4. From the above described schemes is evident that interactions and combinations of thermal and photochemical reactions can affect or regulate the products **A^1^**, **D^1^** and **B^1^** or **D^2^** and **A^2^** equilibrium abundance. The 1,8-naphthalimide fragment electron acceptor property increases the studied hydrazones hydrogen NH acidity to such an extent that in DMF at F^−^ concentration comparable to the equivalent concentration the tautomeric equilibrium is stabilized immediately after the F^−^ions addition in favour the form **A^1^** and/or **D^1^**, **A^2^** and/or **D^2^** respectively. The UV-Vis spectrum is reversibly changed by this altered system irradiation with the wavelength 624 nm and 465 nm (Figure 21).

**Figure 22:**
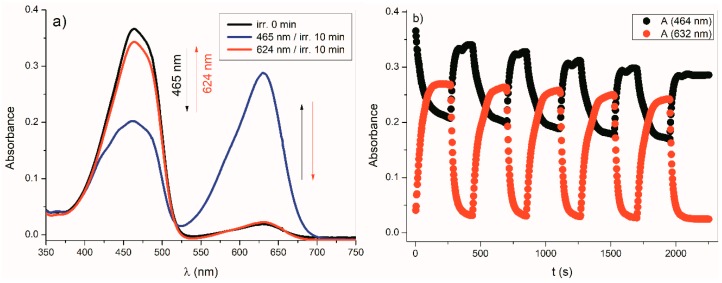
Compound **1** (1 × 10^−5^ mol·dm^−3^) UV-VIS spectra change at the presence of TBABr (1 × 10**^−^**^2^ mol·dm^−3^) (**a**) during alternating light irradiation with the wavelength at 465 nm or 624 nm in DMF (**b**) kinetic recording.

The system has the molecular ON/OFF switch properties. Photochemical switching respectively the range of light-induced colorimetric changes depend on the F^−^ ions concentration in the solution the solvent used and the hydrazone structure. In the given solvent there exist a relatively wide F^−^ ions concentration range (0.3 to 10 equivalents of TBAF) in which the logic 1 and 0 can be defined by colorimetric change with the certain and good resolution. This concentration range depends on the hydrazone structure as well as on the solvent properties. For all studied compounds with the F^−^ ions high excess (about 100 equivalents) in DMF the UV-Vis spectrum of the hydrazone-TBAF system does not longer subject to the described reversible change. At a given light intensity that we have used as the stimulus it is not possible to carry out the photochemically induced tautomeric change. With such a high TBAF concentration excess hydrazone NH hydrogen is cleaved to form structure **A^2^**. The HF_2_^−^ presence was proved by NMR spectroscopy. The structure thermal change **A^2^** to **D^2^** causes absorption band hypsochromic shift at 630 nm about 15 nm (Figure 15). Formed salt has no longer the ON/OFF switch properties. Using the light stimulus, it is not possible to change the achieved tautomeric equilibrium and to achieve the colorimetric response. By addition of water or other dielectrics (e.g., TBABr) it is possible to restore the functionality in this ON/OFF system. Interesting is the TBABr effect on the studied compounds ON/OFF functionality (Figure 22). The TBABr presence unlike TBAF shifts the tautomeric equilibrium towards the hydrazone form (Figure 17). 

**Figure 23:**
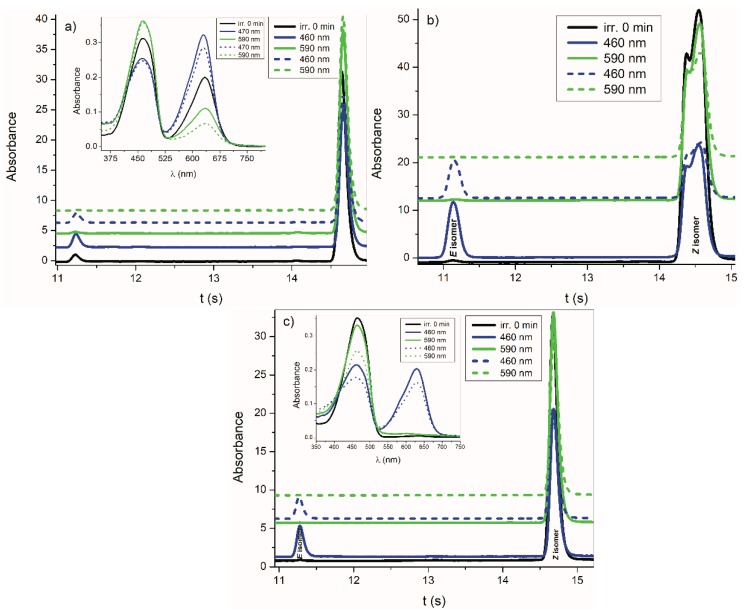
Reaction mixtures HPLC records after alternating light irradiation of compound **1** with the wavelength at 465 nm or 624nm; (**a**) 1 × 10^−5^ mol·dm^−3^ compound **1** + 1 × 10^−5^ mol·dm^−3^ TBAF in DMF; (**b**) 5 × 10^−5^ mol·dm^−3^ compound **1** + 2 × 10^−4^ mol·dm^−3^ TBAF in CHCl_3_; (**c**) 1 × 10^−5^ mol·dm^−3^ compound **1** + 1 × 10**^−^**^2^ mol·dm^−3^ TBABr in DMF (in set: corresponding UV-Vis spectral changes).

If we alternately irradiate the system with the wavelength 465 nm and 624 nm, it has the molecular ON/OFF switch properties. But depending on the irradiation cycles number it is probable that the photochemical reaction takes place. This reflects in the response signal intensity gradual decrease (Figure 22). The HPLC and UV-Vis records (Figure 23) show that after hydrazones irradiation in CHCl_3_ and DMF with the wavelength 460 nm the *E*-isomer is formed in addition to **A^1^**. Structures **A^1^** and **D^1^** cannot be monitored by HPLC. These are converted to **B^1^** during column separation. Subsequent reaction mixture irradiation (624 nm) the reaction products are converted to **B^1^**. The system “hydrazone-TBAF” and “hydrazone-TBABr” ON/OFF switches differ. They have an inverse start at optimal ion concentration. 

**Figure 24:**
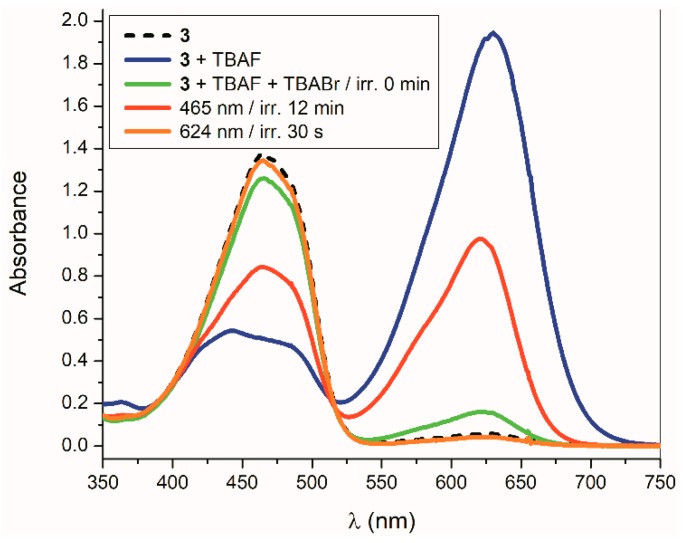
Compound **3** (5 × 10^−5^ mol·dm^−3^) UV-VIS spectra change at the presence of TBAF (5 × 10^−5^ mol·dm^−3^) and TBABr (1 × 10**^−^**^2^ mol·dm^−3^) after alternating light irradiation with the wavelength at 465 nm and 624 nm, in DMSO.

The switch with TBAF requires so as the switching “start” with the light with wavelength at 624 nm. On the contrary the switch with TBABr must “start” with the light with wavelength at 465 nm. In one case, the switch has a logic value of 1 in its initial state and in the second case a logical value of 0. Similar or the same effect on ON/OFF switch functionality has also the TBABr addition to the TBAF hydrazone system (Figure 24). 

**Figure 25:**
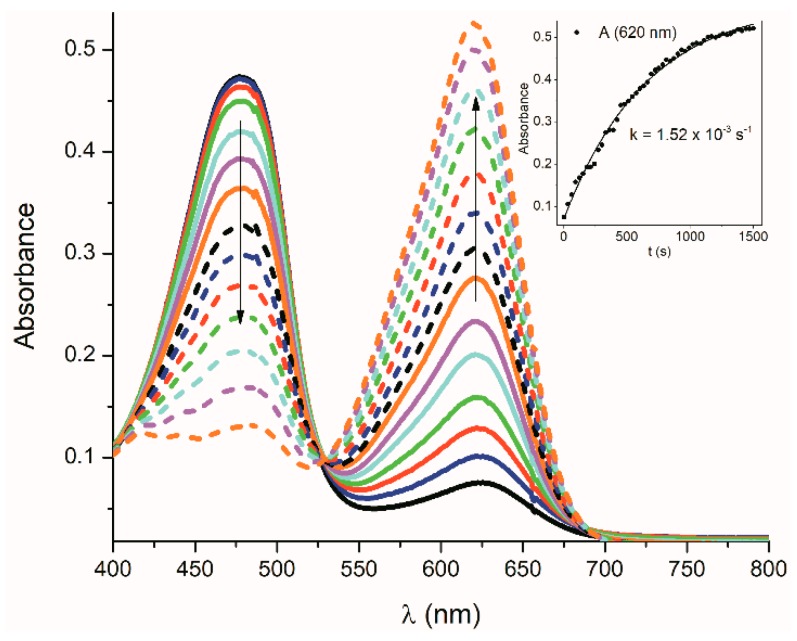
Compound **1** (5 × 10^−5^ mol·dm^−3^) thermal kinetics (25 °C) in the presence of TBAF (1 × 10**^−^**^2^ mol·dm^−3^) in CHCl_3_.

From all studied hydrazones only for compound **5** we did not observe the **A^2^** to **D^2^** thermal change (Scheme 4). We assume that the **D^2^** formation hinders the spatial demands of the 1,8-naphthalimide and 4-dimethoxyphenyl attached to isatin fragment in the structure. Photochemically, this process also takes place at compound **5**, but structure **D^2^** thermally returns to **A^2^** again. Solvents depending on how they affect the tautomeric equilibrium can be divided into two groups. The first group consists of the solvents such as DMF, DMSO and CH_3_CN, which affect tautomeric equilibrium in the same direction as F^−^ ions. They increase the tautomeric reaction rate. The equilibrium state is reached immediately after dissolution of the compounds in these solvents. They stabilize tautomeric forms **A** and **D**. The second group consist of solvents, e.g., CHCl_3_, which, on the contrary, decrease the tautomerisation rate. They shift the tautomeric equilibrium to the hydrazone form. In CHCl_3_ this tautomeric equilibrium rate is significantly slowed as compared to the rate in DMF, DMSO and CH_3_CN. E.g. the rate of tautomerization of compound **1** in CHCl_3_ at 25 °C is equal to k_25 °C_ = 1.52 × 10 ^−3^ s^−1^. Arrhenius activation energy for this compound is ∆E_Arh_ = 53.2 kJ·mol^−1^ (Figure 25). In this case the thermal rate of the tautomeric forms **A^1^** and/or **D^1^** or **A^2^** and/or **D^2^** is slower than the photoreaction rates the product of which is the hydrazone form. 

**Figure 26:**
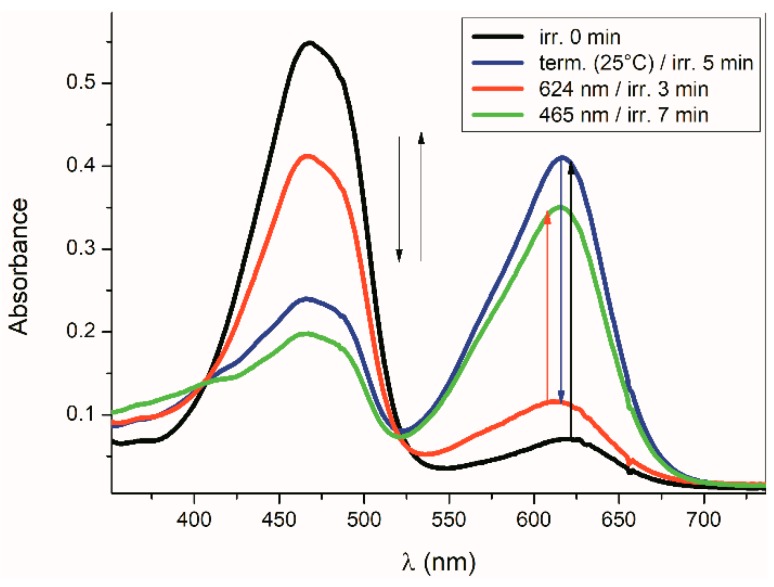
Compound **1** (5 × 10^−5^ mol·dm^−3^) UV-VIS spectra change at the presence of TBAF (2 × 10^−4^ mol·dm^−3^) after light irradiation with the wavelength at 465 nm and 624 nm in CHCl_3_.

Therefore, we can “return” the tautomeric equilibrium to the hydrazone form by a light stimulus at given intensity (624 nm) (Figure 26). From Figure 26 is evident that the hydrazones are in CHCl_3_ not photochemically stable. 

**Figure 27:**
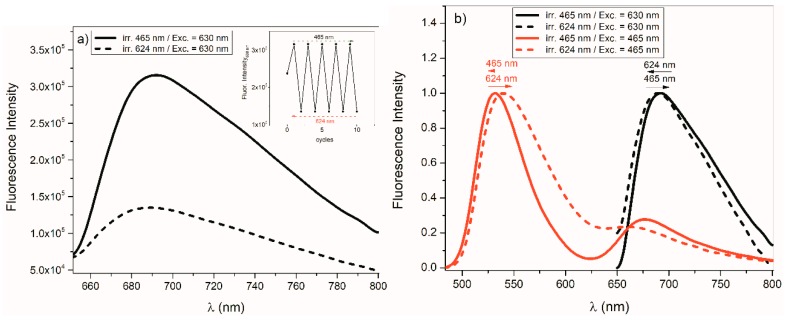
Fluorescence spectrum change (λ_exc._ = 630 nm) alternately irradiated compound **3** (1 × 10^−4^ mol·dm^−3^) with wavelength light at 465 nm a 624 nm in DMF (**a**) normalised fluorescence spectra (λ_exc_ = 465 nm or 630 nm) of compound **3** after λ_exc._ =465 nm or 630 nm in DMF (**b**).

These results however show that the conditions selection make it is possible to affect the tautomeric reaction rate as well as the system stability and thus restore or revive its ON/OFF functionality. Of all previously studied isatin hydrazones only isatin 1,8-naphthalimide hydrazones allowed us to monitor the response to the stimulus by fluorescence. This is caused by both the fluorescence quantum yield but also by the fact that both tautomeric forms exhibit fluorescence and that the tautomeric forms **A** and **D** can be excitated selectively independently from the hydrazone forms (Figure 27). In Figure 27 fluorescence spectrum changes after excitation at 630 nm in the alternating irradiation of 3 with the wavelength at 465 nm and 624 nm can be seen. This ON/OFF functionality corresponds to the tautomeric form **B**, **C** to form **A** or **D** reversible change. At 465 nm excitation, the system behaves equally, while irradiating the sample with light at to the molecule geometry change but mainly it is caused 624 nm, leads to the fluorescence band bathochromic shift at 550 nm. This maximum shift is related by the hydrazone form concentration change in the solution or its association. After the sample irradiation the fluorescence intensity is always lower, than the fluorescence intensity using 465 nm light at 624 nm.

## 3. Materials and Methods

### 3.1. General Information

All chemicals used for synthesis were purchased from Sigma-Aldrich (St. Louis, MO, USA). Solvents were dried and purified by standard methods prior to use. The samples were irradiated in the device’s own construction directly in the spectrometer’s cell by means of LEDs (electrical input 30 to 120 mW). Flash chromatography was performed on Merck (Darmstadt, Germany) silica gel 60. 

### 3.2. Synthesis

A mixture of derivatized isatin (0.4 mmol) and hydrazino isoquinoline 1,3-dione (0.4 mmol) in *n*-butanol (40 mL) was heated to reflux for 24 h. The reaction mixture was concentrated, and the residue was washed several times with hot ethanol to a clear solution. Red solids were isolated.

*2-(2-Propyl)-6-[N’-(2-oxo-1,2-dihydroindol-3-ylidene)-hydrazino]-benzo[de]isoquinoline-1,3-dione* (**1**, >99 %, 0.16 g): ^1^H-NMR (300 MHz, DMSO): δ 14.03 (s, 1H, = NNH), 11.38 (s, 1H, NH_is_), 8.53 (m, 2H), 8.29 (d, 1H, *J* = 8.1 Hz), 7.99 (m, 2H), 7.68 (d, 1H, *J* = 7.4 Hz), 7.35 (d, 1H, *J* = 8.2 Hz), 7.14 (d, 1H, *J* = 7.4 Hz), 6.99 (d, 1H, *J* = 7.3 Hz), 5.40–5.22 (m, 1H, CH), 1.52 (d, 6H, CH_3_, *J* = 6.8 Hz). ^13^C-NMR—due to its extremely low solubility, it was not possible to measure it. Elem. anal. calcd. for C_23_H_18_N_4_O_3_: C 69.34, H 4.55; N 14.06. found: C 59.96; H 4.14; N 23.16. IR (ATR) ν/cm^−1^: 3170, 2961, 1687, 1567, 1357, 786, 733. m. p.: 287–289 °C.

*2-Amino-6-[N’-(2-oxo-1,2-dihydro-indol-3-ylidene)-hydrazino]-benzo[de]isoquinoline-1,3-dione* (**2**, 97%, 0.14 g): ^1^H-NMR (300 MHz, DMSO): δ 14.07 (s, 1H, =NNH), 11.38 (s, 1H, NH_is_), 8.54–8.60 (m, 1H), 8.31–8.35 (m, 1H), 8.06–7.97 (m, 1H), 7.76–7.69 (m, 1H), 7.35–7.43 (m, 1H), 7.12–7.17 (m, 1H), 6.98–7.02 (m, 1H), 5.79 (m, 1H), 3.19 (s, 2H, N(CO)_2_NH_2_). ^13^C-NMR—due to its extremely low solubility, it was not possible to measure it. Elem. anal. calcd. for C_20_H_13_N_5_O_3_: C 64.69; H 3.53; N 18.86; found: C 64.68, H, 3.99; N, 18.88. IR (ATR) ν/cm^−1^: 3314, 3110, 1681, 1648, 1372, 754, 746. m. p.: >300 °C.

*2-(2-Propyl)-6-[N’-(2-oxo-5-trifluoromethoxy-1,2-dihydro-indol-3-ylidene)hydrazino]benzo[de]iso- quinoline-1,3-dione* (**3**, 99 %, 0.19 g): ^1^H-NMR (300 MHz, DMSO): δ 13.99 (s, 1H, =NNH), 11.54 (s, 1H, NH_is_), 8.55 (m, 2H), 8.31 (m, 1H), 8.12 (dd, 1H, *J* = 8.4, 2.4Hz), 7.99 (dd, 1H, *J* = 15.5, 7.3 Hz), 7.68 (m, 1H), 7.36 (d, 1H, *J* = 8.7 Hz), 7.08 (d, 1H, *J* = 8.6 Hz), 5.31 (m, 1H, CH), 1.52 (d, 6H, CH_3_, *J* = 6.9 Hz). ^19^F-NMR (282 MHz, DMSO): δ—57.22 ppm. ^13^C-NMR—due to its extremely low solubility, it was not possible to measure it. Elem. anal. calcd. for C_24_H_17_F_3_N_4_O_4_: C 59.75, H 3.55, N 11.61. found: C 60.02, H 3.49, N 11.60.IR (ATR) ν/cm^−1^: 3221, 1621, 1558, 1132, 777. m.p.: 276–277 °C.

*2-(2-Propyl)-6-[N’-(1-methyl-2-oxo-5-trifluoromethoxy-1,2-dihydro-indol-3-ylidene)hydrazino]-benzo[de]isoquinoline-1,3-dione* (**4**, 99%, 0.19 g): ^1^H-NMR (300 MHz, DMSO): δ 13.95 (s, 1H, =NNH), 8.57–8.50 (m, 1H), 8.39–8.32 (m, 2H), 8.17–8.13 (m, 1H), 7.99–8.02 (m, 1H), 7.74 (m, 1H), 7.46-7.49 (m, 1H), 7.34–7.31 (m, 1H), 5.80 (s, 1H, CH), 3.35 (s, 3H, CH_3_ is), 1.53–1.51 (d, 6H, CH_3_) ppm. ^13^C-NMR—due to its extremely low solubility, it was not possible to measure it. ^19^F-NMR (282 MHz, DMSO): δ—57.22 ppm. Elem. anal. calcd. for C_25_H_19_F_3_N_4_O_4_: C. 60.48, H 3.86, N, 11.29. found: C 60.50, H 3.99, N 11.28. IR (ATR) ν/cm^−1^: 3023, 2931, 1650, 1566, 1099, 775. m.p.: 254–256 °C.

*6-{N’-[4-(2,6-Dimethoxyphenyl)-2-oxo-1,2-dihydro-indol-3-ylidene]hydrazino}-2-(2-propyl)-benzo [de]isoquinoline-1,3-dione* (**5**, 71%, 0.15 g): *E* isomer—^1^H-NMR (300 MHz, DMSO): δ 13.88 (s, 1H, NH), 11.34 (s, 1H, NH), 8.48 (d, 1H, *J* = 7.3 Hz), 8.24 (d, 1H, *J* = 8.3 Hz), 8.15 (d, 1H, *J* = 8.3 Hz), 7.88 (dd, 1H, *J* = 8.3; 7.4 Hz), 7.53 (t, 1H, *J* =8.4 Hz), 7.32 (t, 1H, *J* = 7.7 Hz), 7.00 (d, 1H, *J* = 8.4 Hz), 6.90 (m, 4H), 5.27 (m, 1H, CH), 3.60 (s, 6H, OCH_3_), 1.48 (d, 6H) ppm. ^13^C-NMR (75 MHz, DMSO): δ 164.12, 164.11, 163.63, 157.64, 143.49, 141.58, 133.74, 132.50, 131.42, 130.11, 129.86, 129.10, 127.21, 126.24, 126.15, 123.47, 119.32, 119.00, 116.22, 115.52, 109.97, 108.26, 104.78, 56.04, 44.50, 25.79, 19.99, ppm. Elem. anal. calcd. for C_31_H_26_N_4_O_5_: C 69.65, H 4.90, N 10.48. found: C 70.62, H 4.98; N 10.47. IR (ATR) ν/cm^−1^: 3299, 2945, 1689, 1566, 771, 753. m.p.: 293–295 °C.

### 3.3. Spectroscopic Measurements

NMR spectra were recorded in 5 mm NMR tubes on a Varian NMR System 300 (300 MHz for ^1^H, 75 MHz for ^13^C and 282 MHz for ^19^F) or VNMRS 600 MHz spectrometer (600 MHz for ^1^H; 150 MHz for ^13^C, Varian, Inc., Palo Alto, CA, USA) in DMSO-*d*_6_ and CDCl_3_ as solvents (c_sensor_ = 1 × 10^−4^; anion concentration up to 30 equivalents). Chemical shifts are referenced to tetramethylsilane (TMS) as an internal standard. Attenuated total reflection Fourier transform infrared (ATR-FTIR) spectra of all the described experiments were measured on a Nicolet 6700 FTIR (from ThermoNicolet Corp., Madison, WI, USA). Spectra were recorded with ATR mathematical corrections yielding a 1.0 cm^−1^ actual resolution and 40 measurements were averaged. Spectra for liquid samples were measured in a cell with CaF_2_ windows and a path length of 0.2 mm. Electronic absorption spectra were obtained on a HP 8452A diode array spectrophotometer (Hewlett Packard, Palo Alto, CA, USA). Solution fluorescence was measured in a 1 cm cuvette with an FSP 920 (Edinburgh Instruments, Edinburgh, UK) spectrofluorimeter in a right-angle or front-face arrangement (to exclude solution self-absorption). The solvents used (CHCl_3_, DMSO, DMF) were HPLC (CHCl_3_; LiChrosolv^®^, Merck, Darmstadt, Germany) or UV-spectroscopy grade (DMSO and DMF; Uvasol^®^, Merck) and were used without further purification. Detailed determination of association constant for apparent 1:1 complex is described in ESI Association constant determinations.

### 3.4. HPLC Chromatography

HPLC chromatography was carried out using a chromatographic system (Agilent Technologies, Santa Clara, CA, USA) consisting of a quaternary pump, thermostated column compartment, a diode array detector (VWDG 1314A), manual injector (Rheodyne model 7725i) with 20 μL sample loop, and a degasser (g1379A) all the 1100 series. For all experiments, Column ZORBAXSB- Phenyl (150 mm × 4.6 mm i.d.) was used. For analyses of I, mobile phase A was a methanol/water mixture (ϕr = 1:99) and phase B was acetonitrile. In the analysis of isomers, the isocratic gradient A/B (ϕr = 1:1) at the flow rate of 0.6 mL min^−1^ at 22 °C and detection at 236 nm was used. The injection volume was 20 μL. For analyses of II, mobile phase A was a methanol/water mixture (ϕr = 1:99) and phase B was methanol. In the analysis of isomers, the isocratic gradient A/B (ϕr = 37:13) at the flow rate of 0.6 mL min^−1^ at 22 °C and detection at 236 nm was used. The injection volume was 20 μL.

## 4. Conclusions

Five new isatin 1,8-naphthalimide hydrazones were prepared. These compounds’ UV-VIS and fluorescence spectra are dependent on their concentration in aprotic solvents with a high dielectric constant. By decreasing the hydrazone concentration (c = 1 × 10^−4^ mol.dm^−3^) a new absorption band is formed in the UV-VIS spectra, which maximum depends on the hydrazone structure and is in the 625–650 nm range. The band absorbance increase is accompanied by the band absorbance decrease at 460 nm with isosbestic points maintenance at 518 and 373 nm. At the hydrazone concentration higher than 4 × 10^−5^ mol·dm^−3^ the fluorescence band at 550 nm is quenched and new fluorescence band is formed at 690 nm. Based on NMR measurements it is evident that the studied hydrazones were present in solution in two tautomeric forms. The tautomer’s proportionate representation in aprotic solvents (DMF, DMSO, CH_3_CN) can be reversibly changed by altered irradiation with the wavelength at 465 nm and 624 nm. In addition to the light-stimulated tautomeric equilibrium shift at the irradiation, E⇄Z isomerization occurs. The *E*-isomer in these solvents is thermally unstable. The studied hydrazones irradiation in protic solvents (CH_3_OH) and in non-polar solvents (CHCl_3_ etc.) with low dielectric constant leads exclusively to Z⇄E photochemical isomerization. Studied hydrazones provide coloured solutions in the presence of fluoride, chloride and acetate ions in DMF. The hydrazones sensitivity in the ions presence depend on the bases ionicity. From the data in Table 4 (detection and quantificaton limits) it is evident, that compounds **3** and **4** are considered to be very good F^−^ ion sensors when compared to the previously published data. Anions decrease the hydrazoform concentration that is reflected in the fluorescence spectra by fluorescence intensity decreasing at 550 nm and increasing the second tautomer fluorescence intensity at 690 nm. The studied hydrazones in the presence of fluoride ions have the molecular ON/OFF switch properties with the response in the form of colorimetric, fluorescence changes as the “answer” to the stimulus (light). This ON/OFF function corresponds to the reversible change in tautomeric equilibrium.

## Figures and Tables

**Table 1:** Absorption and fluorescence maxima of isatin 1,8-naphthalimide hydrazones.

Compound	UV-VIS			Fluorescence	
	DMF		CHCl_3_	DMF	CHCl_3_
	λ_1_ (nm)	λ_2_ (nm)	λ (nm)	λ (nm)	λ (nm)
**1 Z**	463	634	464	532/exc.465 686/exc.630	533/exc. 465 -/exc.630
**2 Z**	468	645	468	553/exc.465	-/exc.465
**3 Z**	462	625	464	544/exc.465 688/exc.630	564/exc.465 687/exc.630
**4 Z**	460	625	464	537/exc.465 687/exc.630	556/exc.465 -/exc.630
**5 Z**	474	650 *	468 486 **	532/exc.465 -/exc.630	537,558 **/exc.465 -/exc.630

λ_1_—short-wavelength absorption maximum; λ_2_—long-wavelength absorption maximum; * very weak, ** shoulder.

**Table 2:** The studied hydrazones *Z* and *E* isomers proportional representation in the equilibrium mixture after *Z*-isomers photolysis (465 nm) in CHCl_3_ and DMF determined by HPLC.

Compound	CHCl_3_	DMF
	*Z*/*E*	*Z*/*E*
**1**	1.1	3.5
**3**	3.3	5.9
**4**	16.2	18.1
**5**	1.9	11.4

Compound 2 was not soluble enough.

**Table 3:** Quantum yields and 1,8-naphthalimide hydrazone fluorescence lifetimes; χ^2^—reduced chi-square.

Compound		Φ_F_	τ_1_[ns]	%	τ_2_[ns]	%	τ_3_[ns]	%	χ^2^
**1** 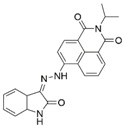	DMF	0.04	0.028	23.5	3.41	48.9	9.02	27.6	1.3
	CHCl_3_	-	0.06	76.9	-	-	7.44	23.1	1.34
	CH_3_OH	0.03	0.04	53.7	3.7	15.7	9.03	30.6	1.4
**2** 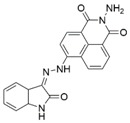	DMF	<0.01	0.04	37	3.2	52.5	10.6	10.5	1.13
	CH_3_OH	<0.01	0.08	27.3	0.98	33.5	6.1	39.1	1.26
**3** 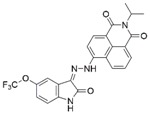	DMF	<0.01	0.03	20. 1	3.51	13.3	8.78	66.6	1.48
	CHCl_3_	-	0.1	59.1	-	-	8.9	40.9	-
	CH_3_OH	<0.01	0.14	61.2	2.87	8.1	8.88	30.7	1.16
**4** 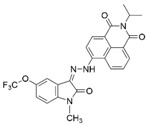	DMF	<0.01	0.12	7.1	4.73	24.7	9.16	68.2	1.03
	CH_3_OH	0.03	0.25	19.9	3.42	18.9	7.89	61.2	1.26
**5** 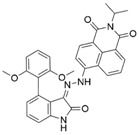	DMF	<0.01	0.07	32.3	-	-	8.2	63.7	1.16
	CHCl_3_	-	0.28	93.4	-	-	8.3	6.6	-
	CH_3_OH	0.02	0.12	73.4	-	-	7.5	26.6	1.18

Φ_F_—Fluorescence quantum yield; τ—Fluorescence lifetime; χ2—Quality of fluorescence lifetime fiting;. %—relative fluorescence intensity of the corresponding component.

**Table 4:** Detection (3σ/S) and quantification (10σ/S) limits (v mol.dm^−3^) of *Z*-izomers isatin 1,8-naphtalimide derivatives for strong basic F^−^ a CH_3_COO^−^ anions in DMF at 298,16 K (based on absorbance at 630 nm; σ = 0,005).

Compound	3σ/S F^−^ (mol·dm^−3^)	10σ/S F^−^ (mol·dm^−3^)	3σ/S CH_3_COO^−^ (mol·dm^−3^)	10σ/S CH_3_COO^−^ (mol·dm^−3^)
**1**	4.7 × 10^−7^	1.6 × 10^−6^	1.8 × 10^−6^	6.0 × 10^−6^
**2**	1.7 × 10^−6^	5.7 × 10^−6^	1.3 × 10^−6^	4.4 × 10^−6^
**3**	3.3 × 10^−8^	1.1 × 10^−7^	2.0 × 10^−7^	6.6 × 10^−7^
**4**	8.3 × 10^−8^	2.8 × 10^−7^	8.5 × 10^−8^	2.8 × 10^−7^
**5**	8.3 × 10^−7^	2.8 × 10^−6^	2.9 × 10^−6^	9.7 × 10^−6^

*σ*—the standard deviation, S is the slope of the *A*_630_ = *f*(*c*_A-_) in the initial linear portion of the curve, where *A*_630_ is the absorbance value at 630 nm and *c*_A-_ is the concentration of the corresponding anion; in all cases *n* = 10.

## Supplementary Materials

The following are available online: compound **1** UV-Vis spectra change during irradiation (λ = 465 nm); Figure S1. Photolysis of compound **2**–**5** in CHCl_3_; Figure S2. The photolysis course of compound **1** to **5** in DMF; Figure S3. TBAF effect on UV-Vis spectra in DMF; Figure S4. ^1^H NMR spectrum (aromatic region) of compound **4** in CDCl_3_; Figure S5. ^1^H NMR spectrum (aromatic region) of compound **4** in DMSO-D_6_; Figure S6.
