# Supplementary material for: Isatin-1,8-Naphthalimide Hydrazones: A Study of Their Sensor and ON/OFF Functionality
Source: Molecules. 2019 Jan 22;24(3):397. doi: 10.3390/molecules24030397 (PMC6385022; doi:10.3390/molecules24030397)
Supplement: Supplementary file 1 [file molecules-24-00397-s001.pdf]

Isatin-1,8-naphthalimide hydrazones: study of their sensor and ON / OFF functionality

Pavol Tisovský*, Miroslav Horváth, Klaudia Csicsai, Jana Donovalová, Juraj Filo, Marek Cigáň, Róbert Sokolík, Gabriela Addová and Anton Gáplovský

Faculty of Natural Sciences, Institute of Chemistry, Comenius University, Ilkovičova 6, Mlynská dolina CH-2, SK-842 15 Bratislava, Slovakia;

mirek.horvath@gmail.com (M.H.); klaudia.jakusova@uniba.sk (K.J.); jana.donovalova@uniba.sk (J.D.); juraj.filo@uniba.sk (J.F.); marek.cigan@uniba.sk (M.C.); robert.sokolik@uniba.sk (R.S.); gabriela.addová@uniba.sk (G.A.) anton.gaplovsky@uniba.sk (A.G.)

* Correspondence: pavol.tisovsky@uniba.sk; Tel.: +421-2-60296-378

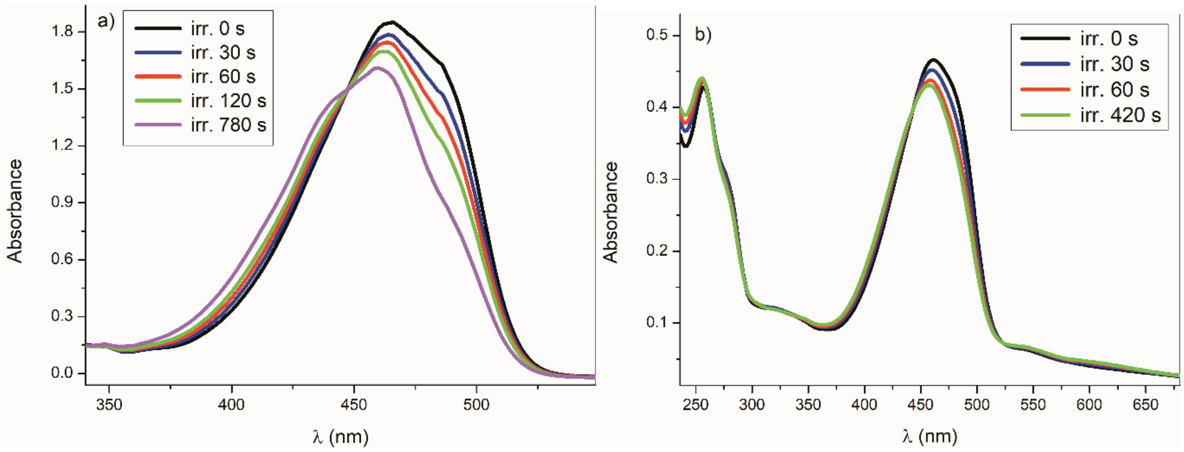

Figure S1. Compound **1** (a) 5 x 10^{-5} mol.dm^{-3} in $CHCl_3$ and b) saturated solution in CH_3OH) UV-Vis spectra change during irradiation (λ=465 nm).

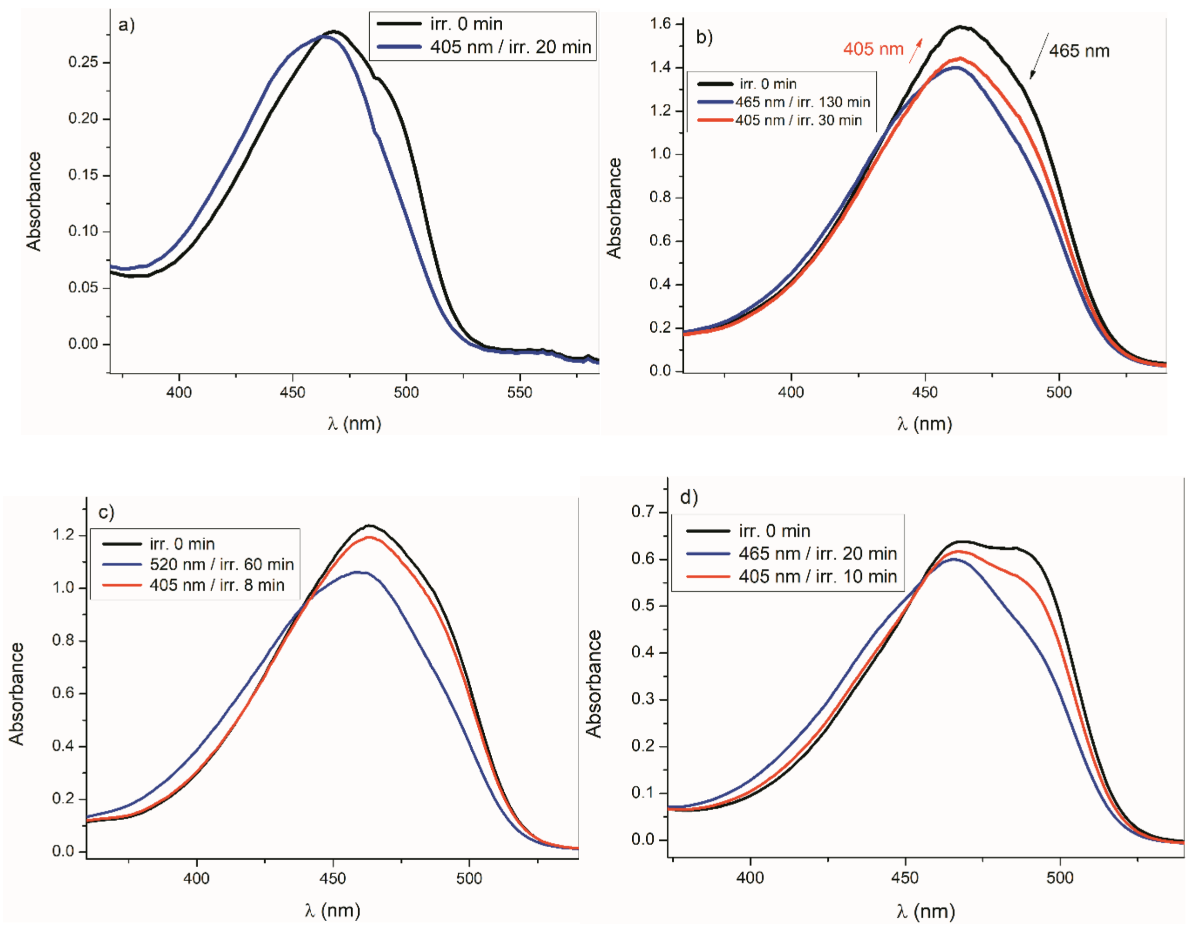

Figure S2. Photolysis of compound **2-5** in $CHCl_3$. a) **2** (saturated solution), b) **3** (5 x 10^{-5} mol.dm^{-3}), c) **4** (1 x 10^{-4} mol.dm^{-3}) and d) **5** (1 x 10^{-4} mol.dm^{-3}).

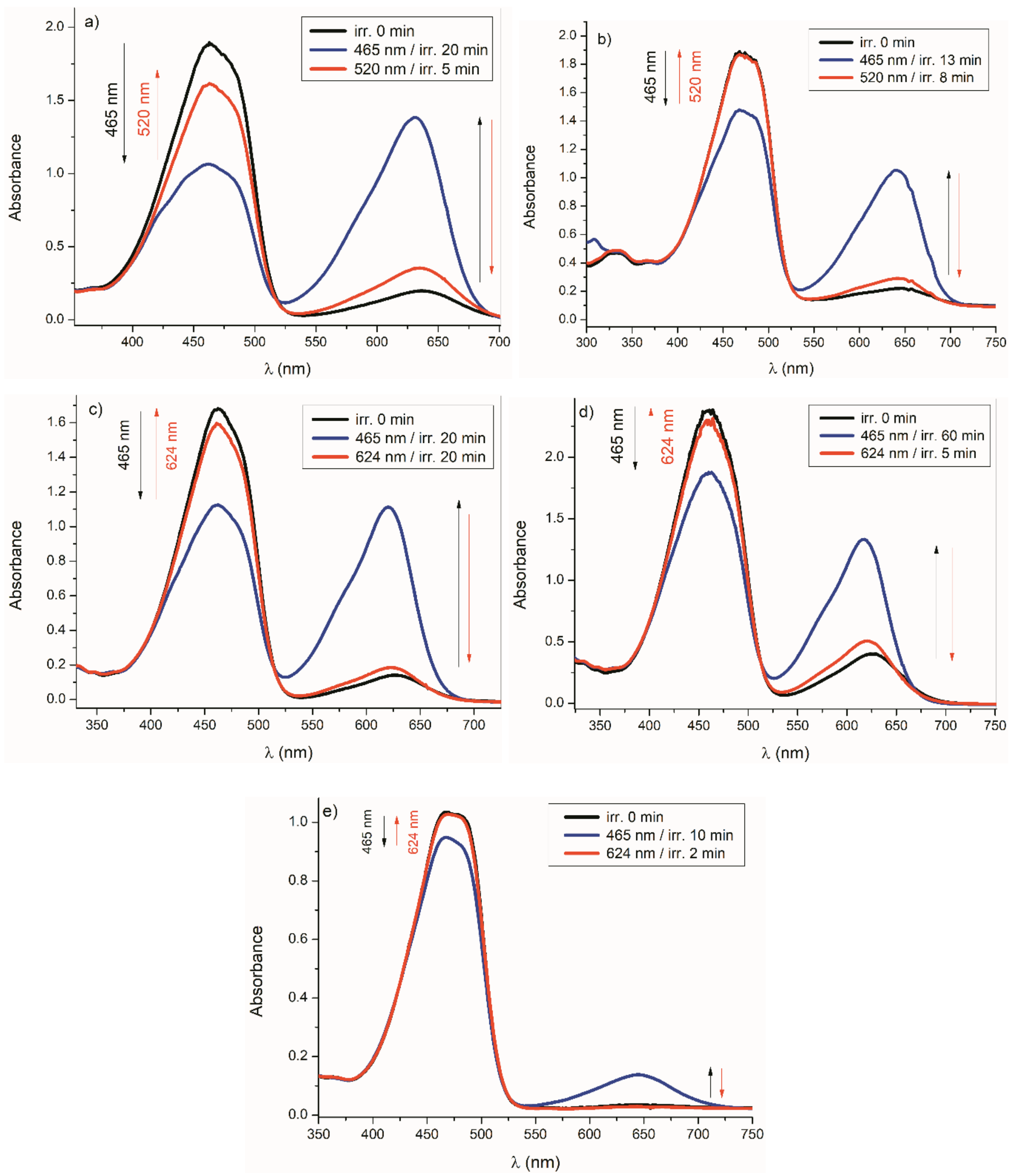

Figure S3. The photolysis course of **1** to **5** in DMF [a) **1** (5 x 10^{-5} mol.dm^{-3}), b) **2** (5 x 10^{-5} mol.dm^{-3}), c) **3** (5 x 10^{-5} mol.dm^{-3}), d) **4** (5 x 10^{-5} mol.dm^{-3}) and e) **5** (1 x 10^{-4} mol.dm^{-3})].

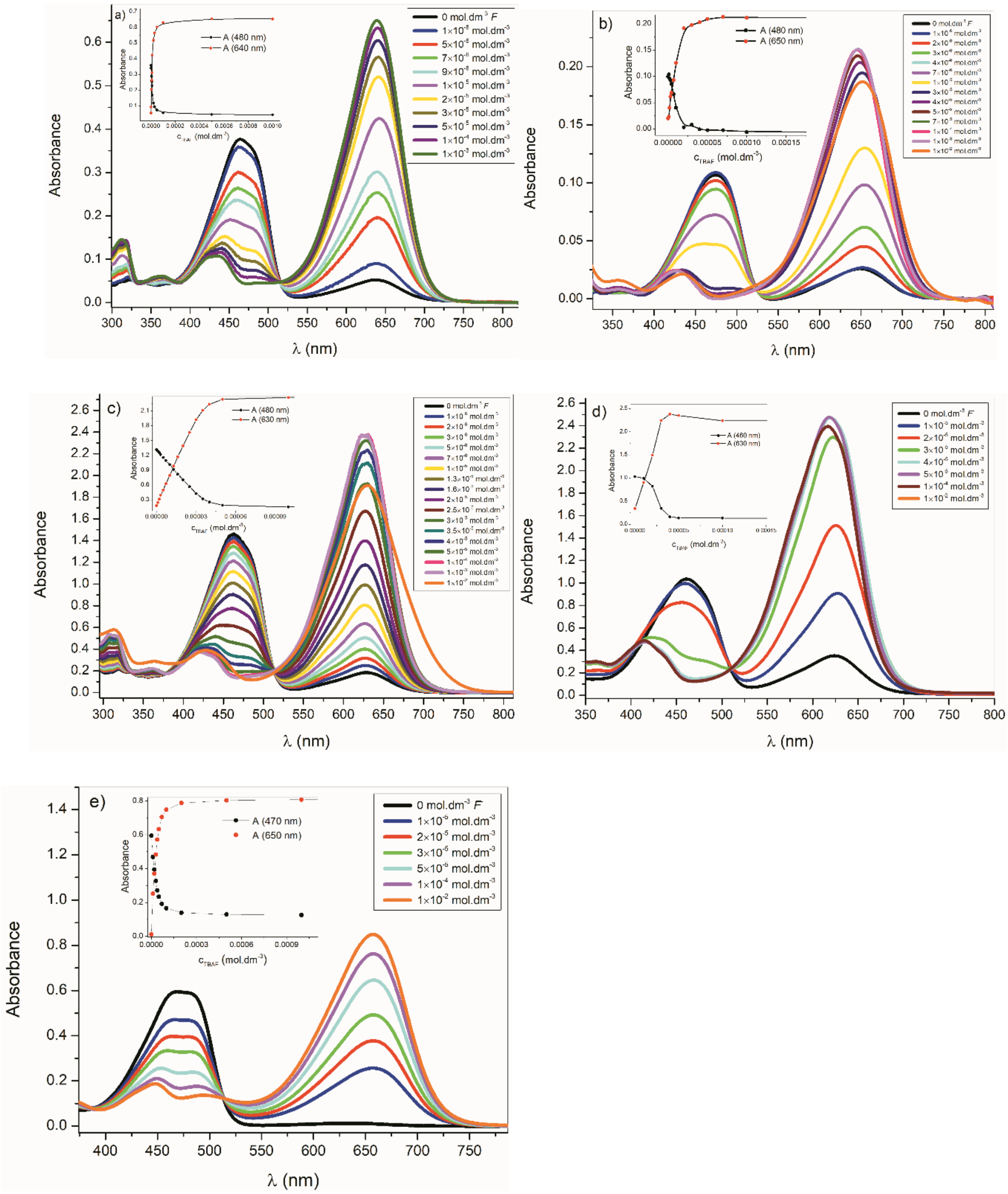

Figure S4. TBAF effect on UV-Vis spectra a) **1** (1 x 10^{-5} mol.dm^{-3}), b) **2** (1 x 10^{-5} mol.dm^{-3}), c) **3** (5 x 10^{-5} mol.dm^{-3}), d) **4** (5 x 10^{-5}mol.dm^{-3}) and e) **5** (1 x 10^{-4} mol.dm^{-3}) in DMF.

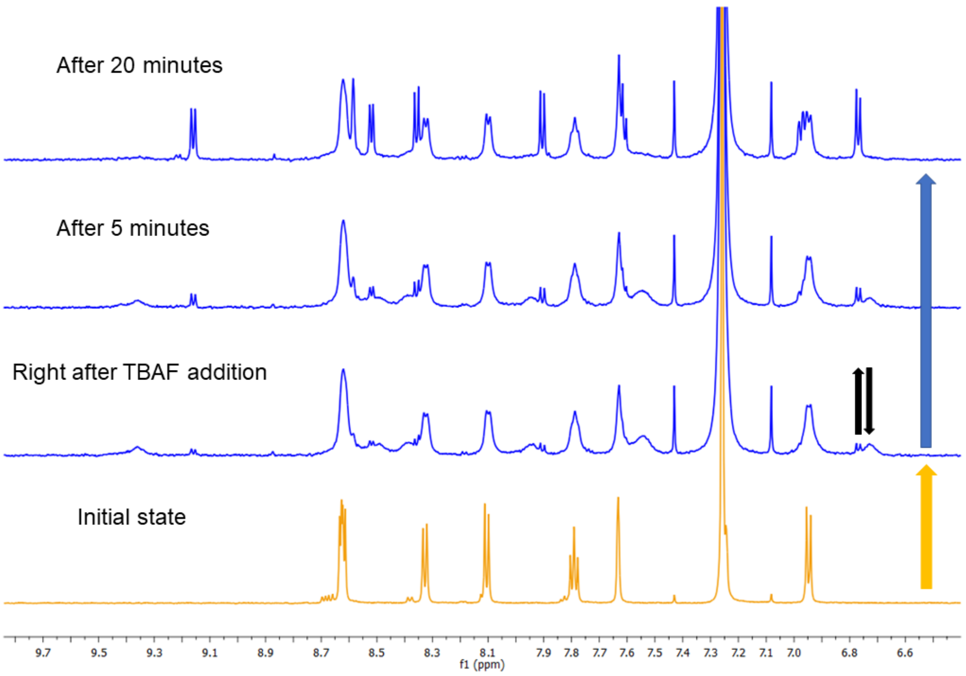

Figure S5: ^{1}H NMR spectrum (aromatic region) of compound **4** in $CDCl_3$: (Orange line) initial state; (blue lines) geometrical changes observed after addition of 1 equivalent of TBAF.

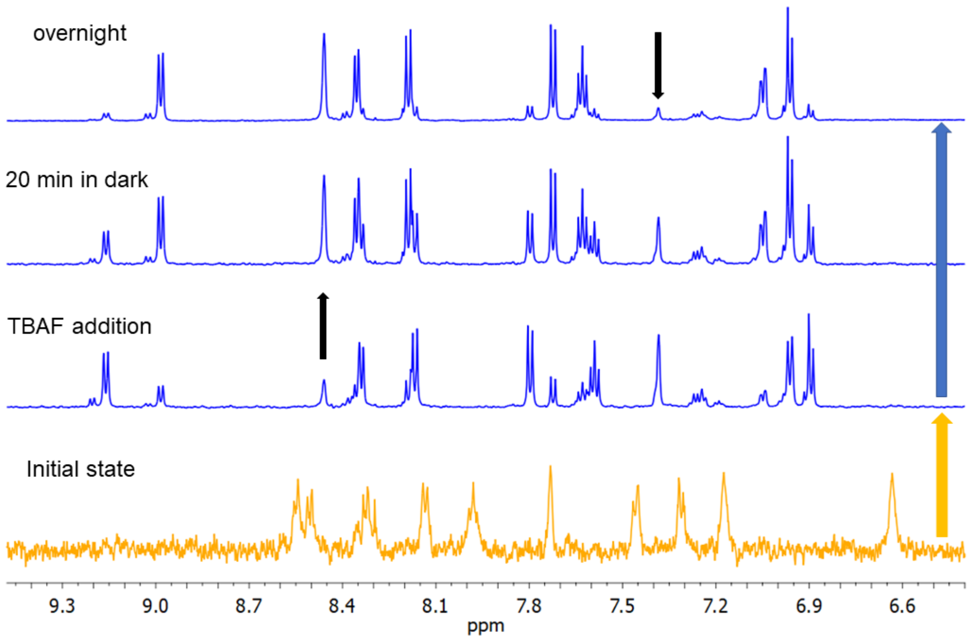

Figure S6: ^{1}H NMR spectrum (aromatic region) of compound **4** in DMSO-D_6: (Orange line) initial state; (blue lines) geometrical changes observed after addition of 2 equivalents of TBAF.

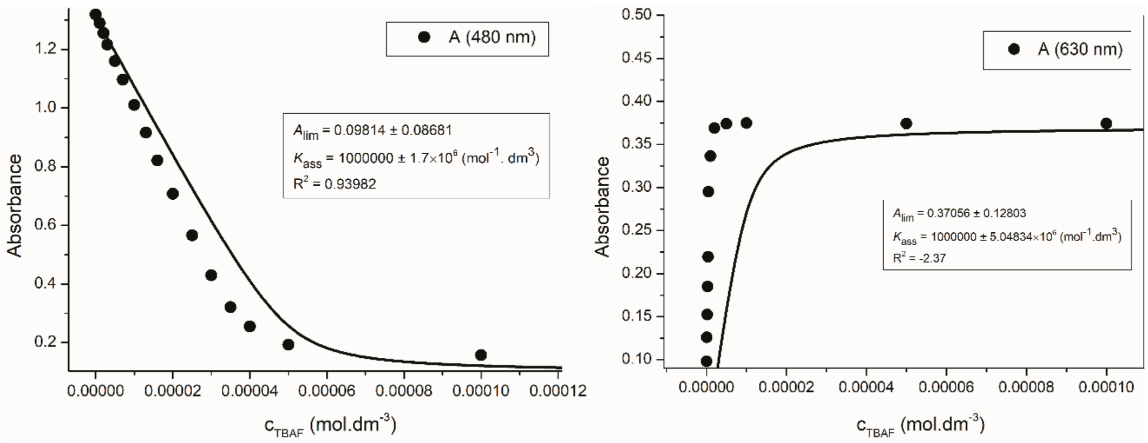

Figure. S7 Titration of **3** by TBAF in DMF and the mathematical fitting of titration data using the equation (S1) (see below).

Association constant determinations

Association constants K_{ass} for apparent isatin 1,8-naphthalimide hydrazones: anion 1:1 complex formation was determined by the acknowledged formula describing complex anion concentration.

$$A = A_0 + \frac{A_{lim} - A_0}{2c_0}\left[c_0 + c_{A^-} + 1/K_{ass} - \left[(c_0 + c_{A^-} + 1/K_{ass})^2 - 4c_0 c_{A^-}\right]^{1/2}\right], \qquad (S1)$$

where: A_0 is the absorbance of free isatin 1,8-naphthalimide hydrazones, A is the isatin pentafluorophenylhydrazone absorbance measured after anion addition, A_{lim} is the isatin pentafluorophenylhydrazone absorbance measured with excess of the particular anion, c_0 is the overall concentration of isatin pentafluorophenylhydrazone and c_{A^-} is the overall concentration of the added anion A^-.

Equation (S1) was rewritten to the following form for nonlinear fit in OriginPro 8.1 software:

$$A = A_0 + c_1 * (\mathrm{P1} - A_0) * (c_0 + \mathrm{x} + 1/\mathrm{P2} - \sqrt{(c_0 + \mathrm{x} + 1/\mathrm{P2})\wedge 2 - 4 * c_0 * \mathrm{x}}), \qquad (S2)$$

where: $c_0 = 1\times10^{-4}$, $c_1 = 1/2c_0 = 5\times10^{3}$, parameter P1 = A_{lim}, parameter P2 = K_{ass} and x = c_{F^-}. The A_0 value was fixed to the absorbance A value for x = 0.
